# Modelling Anti-Ov16 IgG4 Antibody Prevalence as an Indicator for Evaluation and Decision Making in Onchocerciasis Elimination Programmes

**DOI:** 10.1371/journal.pntd.0005314

**Published:** 2017-01-23

**Authors:** Yvonne L. Lont, Luc E. Coffeng, Sake J. de Vlas, Allison Golden, Tala de los Santos, Gonzalo J. Domingo, Wilma A. Stolk

**Affiliations:** 1 Department of Public Health, Erasmus MC, University Medical Center Rotterdam, Rotterdam, The Netherlands; 2 PATH, Seattle, Washington, United States of America; London School of Hygiene and Tropical Medicine, UNITED KINGDOM

## Abstract

**Background:**

Onchocerciasis is targeted for elimination in Africa through annual or biannual ivermectin mass drug administration (MDA). An immunodiagnostic test, based on the detection of human IgG4 antibodies in the blood to the *Onchocerca volvulus*-specific antigen Ov16, is one of the recommended tools for determining whether transmission is interrupted and mass treatment can stop. For different transmission settings, the relationship between post-MDA Ov16 antibody prevalence in children (measured 1 year after the last round of MDA) and the duration and coverage of MDA, the mf prevalence in the population, and the probability that onchocerciasis is eventually eliminated is explored through mathematical modelling.

**Methodology:**

The ONCHOSIM model was extended with new output on the Ov16 antibody serostatus of individuals. Seroconversion was assumed to be triggered by the first worm establishing in the host, with seroconversion occurring either before maturation, after maturation or only after the start of mf production. We are mainly interested in seroconversion rates in children, and for now ignore the possibility of seroreversion to simplify the model.

**Principal findings:**

Yearly repeated MDA leads to a strong reduction in the parasite acquisition rate in humans. This reduces the seroconversion rate in newborns and young children, while those who seroconverted before the start of control remain antibody positive. Both the microfiladermia prevalence in the population aged 5 years and above and the Ov16 antibody prevalence in children under 10 declined with increasing duration of MDA. The association between either of these indicators and the model-predicted probability of elimination was not influenced much by the assumed treatment coverage levels, but was found to depend on baseline endemicity levels, assumptions regarding the trigger of seroconversion, and diagnostic test characteristics (sensitivity and specificity).

**Conclusions:**

Better understanding of the dynamics of Ov16 antibody responses is required for accurate interpretation of seroprevalence data and more precise estimation of endpoint for MDA. Our study demonstrates that this endpoint will be dependent on baseline endemicity levels, which should be taken into account in guidelines for defining when to stop MDA.

## Author Summary

Onchocerciasis is targeted for elimination in Africa through annual or biannual ivermectin mass drug administration. The elimination target places high demands on monitoring and evaluation systems, for timely detection of ongoing transmission and possible recrudescence. Guidelines recommend using serological evaluation by Ov16, to determine the presence of IgG4 antibodies to the antigen Ov16 in children of less than 10 years in order to detect exposure to the *O*. *volvulus* parasite. In this paper, we use the mathematical model ONCHOSIM to explore how the post-MDA Ov16 antibody prevalence in children (measured 1 year after the last round of MDA) is related to local transmission conditions, observed trends in infection during mass drug administration the duration and coverage of mass treatment, test characteristics, mf prevalence and likelihood of interruption of transmission. The level of seroprevalence that is indicative of successful elimination was found to depend on local transmission conditions. This should be considered in survey methodology and criteria for defining whether mass drug administration can safely be interrupted. Remaining uncertainties about the dynamics of seroconversion and seroreversion further complicate the interpretation of seroprevalence data and the definition of thresholds.

## Introduction

Onchocerciasis–or 'river blindness'–is a parasitic disease caused by the filarial worm *Onchocerca volvulus*. In 1995, about 37 million people were infected, facing or affected by visual impairment, blindness, skin lesions, and severe itching [1]. The prevalence of infection in Africa has declined gradually over the last decades, thanks to large-scale control programmes. The Onchocerciasis Control Programme in West Africa was operational from 1974–2002 and has largely eliminated the disease as public health problem from 11 West-African countries through vector control and/or mass ivermectin treatment. The African Programme for Onchocerciasis Control (APOC) started in 1995 with the implementation of ivermectin mass treatment in the remaining 19 endemic countries. APOC scaled up gradually to cover nearly all meso- and hyperendemic areas in need of treatment [2]. Following reports of elimination of onchocerciasis in some West African foci [3, 4], APOC shifted its focus from control to elimination [5]. While much progress had been made when APOC closed in 2015 [6], continued effort is needed to reach the goal of elimination.

The call for elimination places high demands on monitoring and evaluation systems, for timely detection of ongoing transmission and possible recrudescence. Traditionally, active infection has been detected microscopically by counting parasite microfilariae (mf) in skin snips (small superficial skin biopsies). Population survey results are typically summarized in terms of microfiladermia prevalence (mf prevalence) and intensity of the infection (community microfilarial load, CMFL [7]). Disadvantages of this diagnostic test are its invasiveness and low sensitivity in areas where infection prevalence and intensity are low [8–11]. Current guidelines strongly recommend to use entomological evaluation by O-150 polymerase chain reaction (pool screen) testing in blackflies and Ov16 serology testing in children less than 10 years of age for assessing whether mass drug administration can safely be stopped or for verifying that elimination has indeed been interrupted after a post-treatment surveillance period [12]. Skin snip microscopy can still be used as an additional tool, but should not be used as sole indicator.

Ov16 is a recombinant *Onchocerca volvulus* antigen to which IgG4 antibodies are produced that can be detected using immunological methodologies [9, 13]. Early chimpanzee studies suggested that IgG antibodies can be detected in the blood 3–12 months before microfilariae appeared [14], but later studies suggest that may not be the case for IgG4 antibodies [15]. Current diagnostics are based on IgG4 detection, which is thought to be more specific [16]. A previously developed rapid-format card test for detection of anti-Ov16 IgG4 [9] is no longer available, but an anti-Ov16 ELISA test has been used routinely in the Americas and some African countries as surveillance tool [17–19]. A new rapid diagnostic test (RDT) for detection of IgG4 against Ov16 has recently been developed as a practical, convenient, and standardized alternative for use in the field [20]. The WHO guidelines acknowledge that the evidence of the usefulness of Ov16 antibody tests for assessing interruption of transmission is still limited [12]. The diagnostic accuracy of Ov16 antibody tests has mostly been assessed in populations with high onchocerciasis prevalence rather than under low-prevalence conditions prevailing at the end-stage of control programmes. The guidelines for now advise against using the RDT, which first needs to be validated more extensively. It is suggested that the guidelines should be revised by 2020 on the basis of new evidence [12].

For the improvement of guidelines for the use of an Ov16 antibody test in defining when to stop MDA, it is important to understand how the post-MDA Ov16 antibody prevalence in children depends on the many factors influencing elimination prospects of onchocerciasis (including local transmission conditions, baseline endemicity, treatment duration and treatment coverage) and how it is associated with other infection indicators, notably the mf prevalence. This can be explored with mathematical models, which provide useful tools for systematically assessing trends in infection indicators during mass treatment, under a range of different conditions and treatment scenarios [21]. Mathematical models of onchocerciasis transmission have been used extensively in the past to study trends in mf prevalence and estimate the required duration of mass treatment to bring mf prevalence below a certain threshold or achieve elimination [22–27]. Hitherto, trends in Ov16 antibody prevalence have not been studied by mathematical modelling.

We extended the previously-developed and frequently-used simulation model ONCHOSIM, to include information on the Ov16 antibody serostatus of simulated individuals. In this paper, we describe the model extension and explore how the Ov16 antibody prevalence in children is related to local transmission conditions, the duration and coverage of mass treatment, test characteristics, mf prevalence and the probability that the infection is eventually eliminated.

## Methods

### The ONCHOSIM model

ONCHOSIM is an established epidemiological mathematical mode**l** for simulating transmission and control of onchocerciasis in a dynamic population [28], developed by Erasmus MC in collaboration with the Onchocerciasis Control Programme in West Africa (OCP). It has been used extensively to support decision making in onchocerciasis control programmes in Africa [29–31, 23, 32–35, 22, 27].

ONCHOSIM is an individual-based model, describing the transmission of onchocerciasis between individuals in a dynamic human population and the life course of and mf production by individual worms within the human hosts. The software tracks changes in infection intensity (number of worms, density of mf in the skin) within human individuals over time. Together, the individuals form a dynamic human population that changes in size and composition over time due to birth and death of individuals. Optionally, the user can specify a maximum population size: a specified, randomly-selected proportion of the population is assumed to out-migrate when the population size exceeds this maximum. Transmission of infection by flies is simulated deterministically, accounting for differences between individuals in the exposure to fly bites (related to age and sex and a personal index representing other factors influencing exposure); due to this exposure heterogeneity, the rate of acquisition of new infections and resulting infection intensity vary between human individuals, as does their contribution to the infection pool in flies.

The model can simulate the impact of mass treatment with ivermectin and vector control on transmission and infection indicators. Epidemiological surveys are simulated to obtain information on the infection status of individuals in the population at specified moments in time (model output). Standard output includes the expected mean mf count in 2 skin snips per individual, summarized at population level in terms of mf prevalence and the community microfilarial load (CMFL) [7]. The model accounts for imperfect sensitivity of the skin snip microscopy method [36, 37] by assuming variation in actual mf counts given an underlying simulated mf density in the skin and the possibility of false-negative mf counts. All individuals in the population are assumed to participate in the surveys.

A detailed formal description of the ONCHOSIM model with JAVA program code is provided elsewhere for version 2.58Ap9 of the model (see additional files 1 and 2 in [27]). For the current study, ONCHOSIM version 2.74 was used, which incorporates extra output concerning the Ov16 serostatus of individuals based on their history of infection, as described below. S1 Appendix provides a complete overview of the probability distributions, functional relationships and parameter values used in this study.

### Modelling Ov16 positivity

The model was extended to generate extra output concerning Ov16 serostatus of individuals based on their history of infection. Seropositivity is described as a binary output, similar to the IgG4-based Ov16 antibody RDT: individuals are considered to be either seropositive or seronegative, and degrees of antibody levels are not considered. It is not exactly known what triggers seroconversion and how long it takes after the trigger for an individual to become seropositive. Therefore, we consider three alternative hypotheses in this study.

Hypothesis 1: Ov16 seroconversion occurs when a single L3 larva successfully settles in the human body to eventually develop into an adult male or female worm. Seroconversion occurs before maturation of the worm. The ONCHOSIM model assumes that 0.31% of inoculated L3 larvae will survive to develop into an adult worm successfully; unsuccessful inoculations do not trigger seroconversion. The ONCHOSIM model assumes a maturation period of 1 year for worms, implying that antibodies are detectable at least 1 year before mf are present, or longer in the absence of a mature male-female worm pair. An early chimpanzee study suggested that seroconversion occurs before the mf are detectable in the skin [14], although this was based on detection of total IgG and this may not necessarily be the case for IgG4 antibodies [15]. Antibody positivity in combination with mf negativity may also occur due to false-negative mf tests at low mf densities or after clearance of infection (before or in the absence of seroreversion).

Hypothesis 2: Ov16 seroconversion occurs when the first male or female worm in the human body has matured; considering the assumed 1-year maturation period of worms, the seroconversion occurs one year later than under hypothesis 1. We assume that seroconversion is independent of the presence of mf. Seroconversion will precede the occurrence of mf in the skin, if seroconversion is triggered by a single worm or single-sex infection, but it can also coincide with the appearance of mf when a male-female worm-pair is present. Antibody positivity in combination with mf negativity may also occur due to false-negative mf tests at low mf densities or after clearance of infection (before or in the absence of seroreversion).

Hypothesis 3: Ov16 seroconversion occurs at the first onset of mf production. Seroconversion therefore requires the presence of at least one male and one female worm, and may occur considerably later than under hypothesis 2. This hypothesis is supported by recent chimpanzee studies, that show that–after the inoculations of several hundreds of L3 –IgG4 seroconversion occurs around the same time as the first occurrence of mf in the skin [15]. Antibody positivity in combination with mf negativity only occurs due to false-negative mf tests at low mf densities or after clearance of infection (before or in the absence of seroreversion).

The model provides output on the proportion of people by age group that has met the criteria for seroconversion (according to the selected hypothesis). This is translated into an expected seroprevalence, adjusting for the assumed sensitivity and specificity of the test. In the model, test sensitivity is defined as the proportion of Ov16 antibody test positives among individuals who have met the criteria for positivity (true positive rate). Field data typically show that 10–30% of mf positives, who by definition have met the criteria for seroconversion in our model, do not have detectable antigen level [13, 9, 20]. This might be due to the following mechanisms, between which our model does not distinguish: 1) some individuals are unresponsive and never mount a detectable antibody response against Ov16 antigen in spite of meeting the criteria for seroconversion; 2) a proportion of people who actually do have Ov16 antibodies are incidentally missed by the test, e.g. if the human antibody levels are low or the test platform does not detect all Ov16 antibodies due to the non-native nature of the antigen presented in the test. The relative importance of these two mechanisms cannot easily be estimated from available data, and for now we do not explicitly distinguish them. Specificity is defined as the proportion of Ov16 antibody test negatives among individuals who have not met the trigger for seroconversion (true negative rate). Sensitivity and specificity are assumed to be independent of an individual’s age, infection status, or history of infection.

In this paper, we are mainly interested in seroconversion and seroprevalence in children and the impact of interventions on the seroconversion rate. To keep the model simple, we ignore for now the possibility of seroreversion (with the test turning negative after prolonged lack of boosting). We assume that antibodies remain lifelong detectable so that there is no seroconversion, even though antibody concentrations may decline after clearance of infection [15] and in the absence of boosting by new infections,.

### Simulated scenarios

Core model parameters were quantified as described previously, unless a different value is specified below [23]. A brief summary is provided here. The model typically represents the dynamic population of an onchocerciasis-endemic community. The simulated community population is dynamic, but population growth is restricted: whenever the simulated population size exceeds a specified maximum (here set at 440 people, reflecting a typical medium-size village or subcommunity of a larger village), 10% of the population is assumed to out-migrate (randomly selected, permanently removed from the population). Otherwise, the model represents a closed transmission system.

Perennial transmission with a period of moderate transmission (monthly biting rates 25%-42% lower than the yearly average) and a period with high transmission (monthly biting rates 17%-45% higher than the yearly average) is assumed. The overall annual biting rate was varied between scenarios, to simulate settings with different transmission conditions (ABR 9,409; 10,150; 14,098; 18,078; or 22,212). These values were derived by calibrating the ABR to obtain on average the following pre-defined model-predicted baseline CMFL levels: of 5, 10, 30, 55, or 80 mf/skin snip. Thereby our scenarios capture a realistic range of meso, hyper and holo-endemic endemicity levels. This allows us to study how the relationship between post-MDA Ov16 antibody prevalence in children (measured 1 year after the last round of MDA) and probability of elimination depends on transmission conditions. For each transmission setting, the impact of annual ivermectin mass treatment, while varying the duration of annual mass treatment (1, … 25 rounds) and treatment coverage (60%, 70% or 80% of the total population) was simulated. This leads to a total of 375 scenarios considered in the baseline analysis (varying with respect to annual biting rate, duration of annual mass treatment, and treatment coverage).

The probability that simulated individuals participate in mass treatment with ivermectin is governed by age (children under five years of age are not treated; typically around 15% of the population in Africa), sex (the participation rate is somewhat lower in women of reproductive age in view of ineligibility of pregnant and lactating women), and an individual compliance factor (the higher the factor, the higher the probability that an individual participates in any given treatment round). We further assume that 5% of individuals would never participate in treatment (systematic non-compliance, e.g. because of chronic illness or refusal). Under these assumptions the maximum achievable coverage is about 80%. How the overall coverage is calculated is presented in Stolk *et al* [27] (see additional file 1, section 3.4).

Treatments are given just before the start of the high transmission season. We assumed that treatment kills all mf instantaneously; it does not kill adult worms, but treatment does lead to a complete, but temporary, interruption in the production of mf by all female adult worms. Production recovers gradually over time in all worms, reaching maximum production capacity after 11 months on average. The adult female worms’ capacity to produce mf after recovery is irreversibly reduced by 34.9% on average per treatment. Effects of multiple treatments are assumed to be multiplicative. Both the duration of the recovery period and the irreversible reduction in mf production vary stochastically between worms and treatments. Five percent of treatments are assumed ineffective (e.g. because of malabsorption).

In our baseline scenarios we assumed a sensitivity of 80% and specificity of 99% of the antibody detection test. These values were varied in a sensitivity analysis (sensitivity: 70%, 80%, 90%, 100%; specificity 95%, 97%, 99%, 100%) based on published data of the sensitivity and specificity of the Ov16 IgG4 or IgG biomarker against microfilaria status and test positivity in unexposed individuals [16, 13, 14, 9].

### Simulation output and analysis

ONCHOSIM is a stochastic model, and therefore repeated simulations with the same input values will lead to slightly different results. We did 1,000 repeated runs per scenario. Because of chance effects in the warming-up period of a simulation, the introduction of infection does not always lead to a stable endemic situation. Especially when biting rates are low, the infection may by chance go extinct. Therefore, runs with a pre-control mf prevalence in the general population of less than 20% were considered as failed, and these were excluded from further analyses (failure only occurred in scenarios with pre-control CMFL of 5 and 10 mf/ss, in 124 and 3 out of 1,000 runs, respectively).

We simulated epidemiological surveys to obtain output on the simulated mf prevalence, CMFL, and Ov16 antibody prevalence for each of the three hypotheses. The first survey was scheduled at t = 0, just before the first treatment; surveys were repeated annually (always just preceding a next treatment round if applicable) until 50 years after the last treatment. The mf prevalence was assessed for the entire population aged 5 years and above, considering that in real life children under 5 are often excluded from surveys or underrepresented. The CMFL was calculated as the geometric mean of the individual mf counts + 1 in adults aged 20 years and above. The Ov16 antibody prevalence was measured in children aged 0–9, as recommended by the World Health Organization [12], unless stated otherwise; for one analysis we considered a wider range (0–19 years) to investigate how this would change the results. The simulated mf prevalence, CMFL, and Ov16 antibody prevalence were calculated under the assumption that all individuals in the specified age group participate in the epidemiological surveys and diagnostic tests are always performed successfully. The model does account for measurement variation in diagnostic test results (stochastic variation in mf counts with a possibility of false-negative mf counts; possibility of false-positive or false-negative results in antibody tests due to imperfect sensitivity and specificity).

Outcomes of individual runs were averaged by scenario (after exclusion of failed runs). The mf prevalence in the last survey (i.e. 50 years after the last treatment) was used to define whether the simulation had resulted in elimination or not. Elimination was said to occur if mf prevalence measured 50 years after the last treatment round was equal to zero. The probability of elimination was calculated per scenario as the percentage of non-failed runs resulting in elimination.

## Results

**Fig 1:**
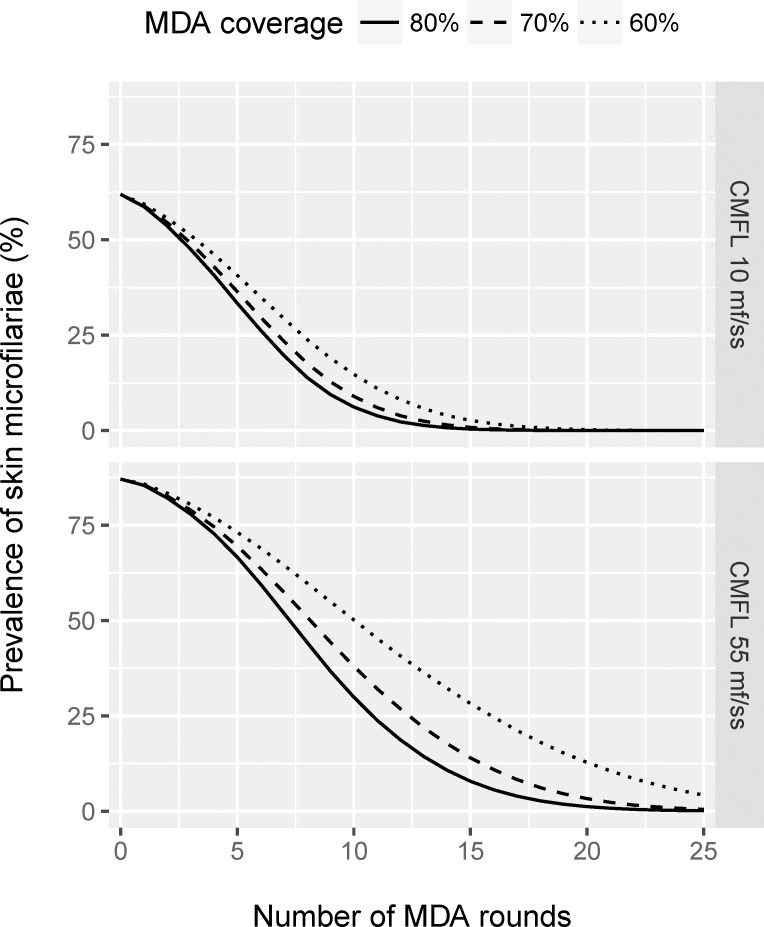
Model-predicted trends in mf prevalence for the population aged 5 years and above during 25 years of annual ivermectin mass treatment, with the first treatment provided at time = 0. Average of 1,000 simulations (minus failed runs) per scenario. Results are shown for transmission settings with moderate and high transmission (ABR 10,150 and 18,078; average pre-control CMFL 10 and 55 mf/skin snip, respectively) and for different treatment coverage levels. The curves connect yearly model-predicted mf prevalence levels, always measured just before a treatment and exactly one year after the previous treatment. For clarity, we do not show trends between these yearly measurements (immediate drop in mf prevalence followed by a gradual increase until the next treatment). See S2 Appendix for a stochastic variant of the figure. The last measurement shown is 1 year after the last treatment.

Fig 1 shows the average model-predicted trends in mf prevalence over time during a 25-year programme of annual ivermectin mass treatment, for transmission settings with moderate and high transmission (ABR 10,150 and 18,078; average pre-control CMFL 10 and 55 mf/skin snip, respectively) and for different levels of treatment coverage. The first round of mass drug administration (MDA) is at t = 0. The figure is based on yearly measurements, always done just before a treatment and exactly one year after the previous treatment. Thus, the figure does not show the immediate drop in mf prevalence directly after treatment and the subsequent increase over time until the next treatment (see other ONCHOSIM-based publications for example of such patterns, e.g. [22, 23]). Multiple annual treatment rounds result in a gradual decline in mf prevalence. A higher coverage causes a faster decline in mf prevalence. The time to reach near-zero mf prevalence levels is longer for the scenarios with higher baseline CMFL levels (due to higher worm burden and more intense transmission) and lower coverage. For clarity, we only show the mean predicted trend in Fig 1 and in other figures below. A stochastic variant of Fig 1 is presented in S2 Appendix.

**Fig 2:**
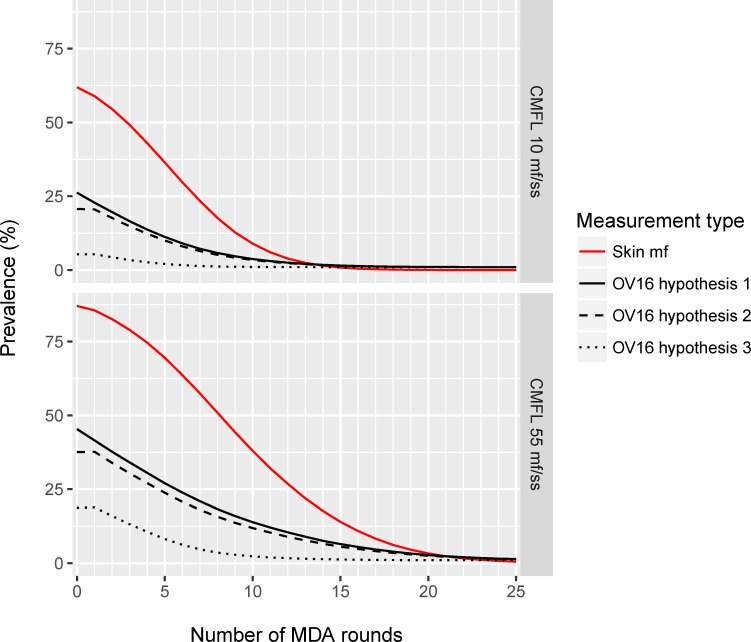
Model-predicted trend in mf-prevalence in the population aged 5 years and above and Ov16 antibody prevalence in children aged 0–9 years in relation to the duration of annual mass drug administration, assuming a fixed coverage of 70%. Average of 1,000 simulations (minus failed runs) per scenario. Results are shown for transmission settings with moderate and high transmission (ABR 10,150 and 18,078; average pre-control CMFL 10 and 55 mf/skin snip, respectively) and for different treatment coverage levels. The Ov16 antibody prevalence was estimated assuming that the Ov16 antibody test has a sensitivity of 80%, and specificity of 99%. Results are shown for each of three hypotheses regarding the seroconversion trigger, with the Ov16 antibody test becoming positive as soon as the first male or female worm establishes in the human body, before it matures (hypothesis 1); idem, but after maturation (hypothesis 2); or after the start of mf production (hypothesis 3).

Average trends in mf prevalence for the scenarios with 70% treatment coverage are shown again in Fig 2, which in addition shows the model-predicted trends in Ov16 antibody prevalence in children aged 0-9 for each of the three different hypotheses about the seroconversion trigger. The decline in mf prevalence results in a lower force-of-infection and declining probability of acquisition of infection in young children. This is reflected by the decline in Ov16 antibody prevalence in children. The Ov16 antibody prevalence never reaches zero, because the assumed specificity of the test was 99% (assumed sensitivity was 80%). The level of Ov16-antibodies depends on the assumed trigger: the predicted Ov16 antibody prevalence levels are considerably lower for hypothesis 3 (seroconversion triggered by the start of mf production) than for hypothesis 1 or 2 (seroconversion is triggered by the first male or female worm establishing in the human body, directly at entry or after its maturation (hypothesis 2). The difference between hypothesis 1 and 2 is small. A stochastic variant of Fig 2 is presented in S2 Appendix.

**Fig 3:**
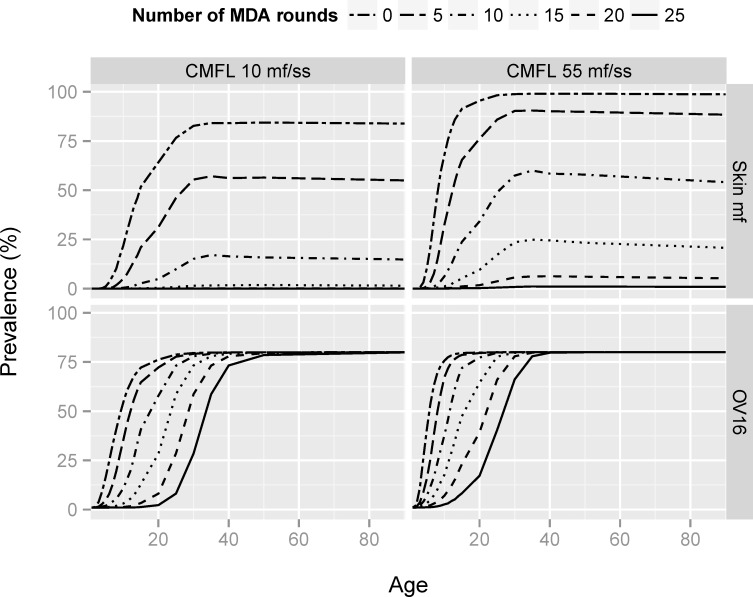
Model-predicted age patterns of mf and Ov16 antibody prevalence in relation to the number of mass treatment rounds provided, assuming that treatment coverage is 70%. Average of 1,000 simulations (minus failed runs) per scenario. Results are shown for transmission settings with moderate and high transmission (ABR 10,150 and 18,078; average pre-control CMFL 10 and 55 mf/skin snip, respectively) and for different treatment coverage levels. The Ov16 antibody prevalence was estimated according to hypothesis 2, assuming that the Ov16 antibody test has a sensitivity of 80% and specificity of 99%, and that seroreversion does not occur.

Fig 3 shows how age patterns of mf prevalence and Ov16 antibody prevalence change after prolonged mass treatment. The shape of the age-mf prevalence curves remains unchanged (showing an increase in mf prevalence with age until it stabilizes at its maximum achieved around the age of 30), but the maximum level is reduced to lower levels with increasing duration of mass treatment. The curve of Ov16 antibody prevalence by age shifts to the right with increasing duration of mass treatment. The maximum Ov16 antibody prevalence remains the same after prolonged ivermectin mass treatment, but eventually this level is only seen in the highest age groups. These patterns result from the assumption that all those who seroconverted before the start of control remain antibody positive (no seroreversion), while the seroconversion rate for newborns and those previously uninfected progressively declines because the force-of-infection is reduced due to mass treatment. Uncertainty in these model assumptions is further discussed below.

**Fig 4:**
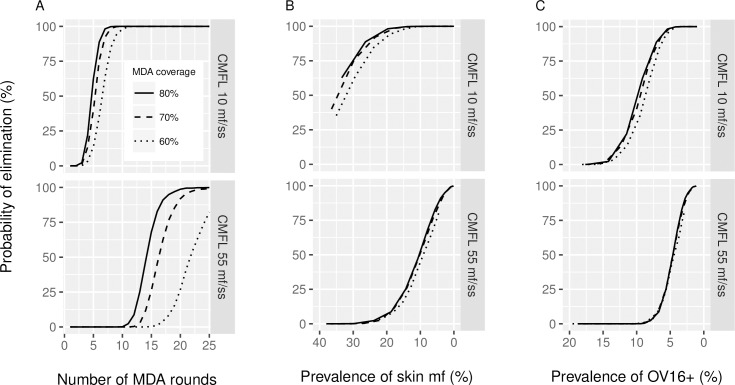
Probability of elimination in relation to the duration of mass treatment and the one-year post-treatment mf prevalence or Ov16 antibody seroprevalence. A) The probability of elimination in relation to treatment duration, assuming that treatment would be discontinued after the indicated treatment duration. B) Probability of elimination in relation to the post-MDA mf prevalence in the population aged 5 years and above, measured one year after 1, 2, 3, … 25 treatment rounds, assuming that no further treatments take place. The lines connect outcomes for different durations of MDA, for the indicated coverage levels (60%, 70%, or 80%). C), similar to B, with probability of elimination shown in relation to the post-MDA Ov16 antibody prevalence in children aged 0–9, measured one year after the 1, 2, 3, … 25 treatment rounds. The Ov16 antibody prevalence was estimated according to hypothesis 2, assuming that the Ov16 antibody test has a sensitivity of 80%, and specificity of 99%. Note that the values on the X-axis in B and C is sorted from highest to lowest, for comparability with A. Results are shown for transmission settings with moderate and high transmission (ABR 10,150 and 18,078; average pre-control CMFL 10 and 55 mf/skin snip, respectively). See Fig 2 for the information on the mean mf and Ov16 antibody prevalence in relation to the duration of MDA.

In Fig 4A, the probability of elimination is shown in relation to the duration of treatment, baseline CMFL and treatment coverage level. The elimination probability gives the probability of eventually finding zero mf prevalence 50 years after the indicated number of MDA rounds, if MDA is discontinued after the indicated number of rounds. The same data are presented in Fig 4B and 4C, but with the probability of elimination plotted against the mf prevalence (in the population aged 5 years and above) and Ov16 antibody prevalence (in 0–9 year olds) (hypothesis 2), respectively, as measured one year after each treatment duration. See Fig 2 for the association between these infection indicators and the duration of MDA. The association between the mf or Ov16 antibody prevalence one year after treatment and the probability of elimination is not influenced much by the achieved coverage levels, but does depend on baseline endemicity levels, which is a proxy for the local transmission intensity potential.

**Fig 5:**
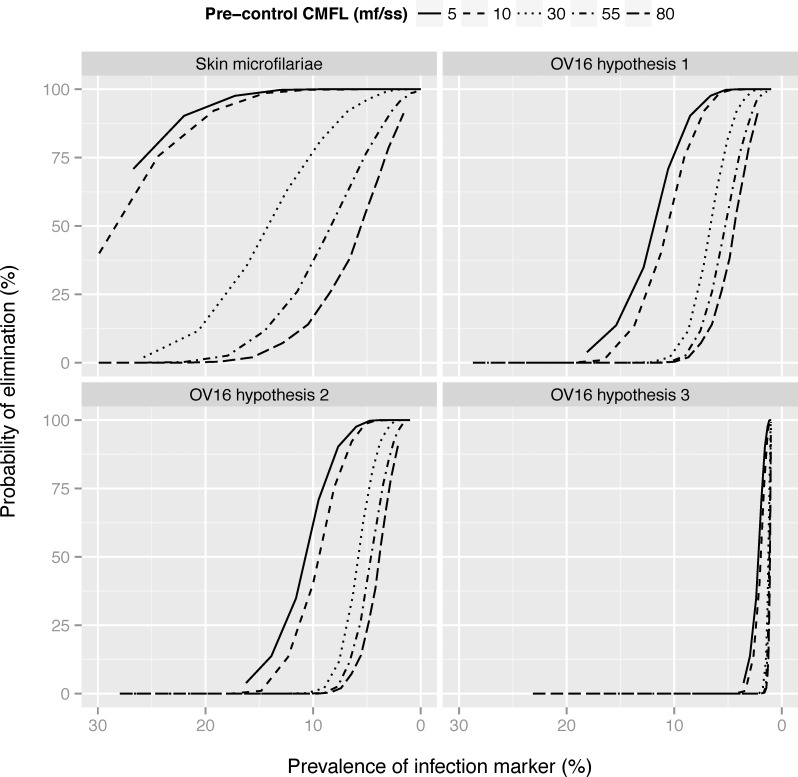
Probability of elimination in relation to the average post-MDA mf prevalence or Ov16 antibody prevalence by pre-control CMFL. The mf prevalence was assessed in the population aged 5 years and above and Ov16 antibody prevalence in children aged 0–9 (hypothesis 1–3), one year after 1, 2, 3, … 25 treatment rounds with a treatment coverage of 70%. The probability of elimination was assessed after each duration, assuming that treatment would be discontinued thereafter. The separate lines connect outcomes on different treatment durations for a given transmission setting and baseline CMFL. The Ov16 antibody prevalence was estimated assuming that the Ov16 antibody test has a sensitivity of 80%, and specificity of 99%. Note that the horizontal axis is ordered from highest to lowest.

The impact of baseline endemicity or local transmission intensity potential is brought out more clearly in Fig 5. As shown in Fig 2, about 15 rounds of MDA are required to bring the mean Ov16 antibody prevalence in the age group 0–9 (according to hypothesis 2) down to 5% in a high transmission setting with ABR 18,078 and average CMFL at baseline 55 mf/snip as example. This duration and resulting mean Ov16 antibody prevalence are associated with about 40% probability of elimination. In sites with moderate transmission (with ABR 10,150 and average CMFL at baseline 10 mf/snip), the same mean Ov16 antibody prevalence is reached after 8 rounds and this corresponds to 95% probability of elimination. An Ov16 antibody prevalence of < = 2% is associated with high probability of elimination in most situations, except perhaps in the areas with very high pre-control endemicity of 80 mf/ss. Similar patterns are seen for Ov16 antibody prevalence under hypothesis 1 or the mf prevalence. The association is different if Ov16 seroconversion is assumed to be triggered by the start of mf production (hypothesis 3). A low Ov16 antibody prevalence among 0–9 year olds is not very informative for assessing the elimination probability under hypothesis 3. At lower values, the slope of the curve is very steep, making it difficult to distinguish situations with 10% or 90% probability of elimination based on this indicator alone.

**Fig 6:**
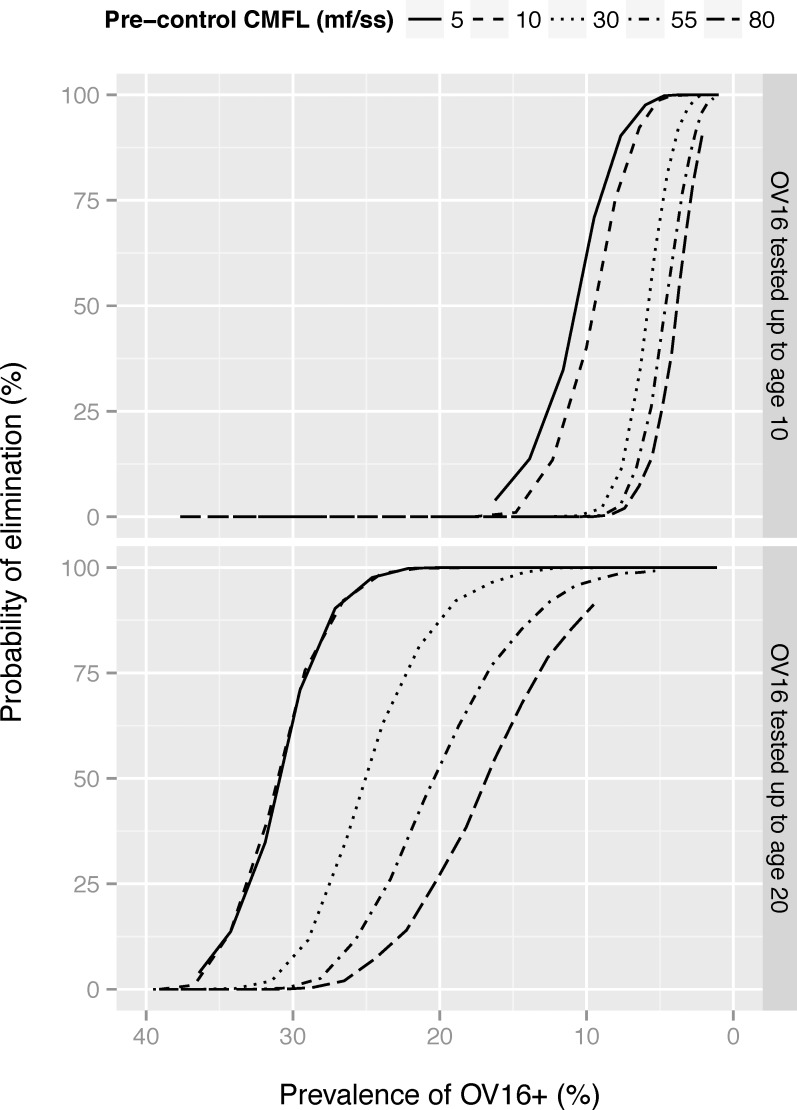
Probability of elimination in relation to the average post-MDA mf prevalence or Ov16 antibody prevalence, by pre-control CMFL and age group tested for Ov16. The mf prevalence was assessed in the population aged 5 years and above and Ov16 antibody prevalence in children aged 0–9 (hypothesis 2), one year after 1, 2, 3, … 25 treatment rounds with a treatment coverage of 70%. The probability of elimination was assessed after each duration, assuming that treatment would be discontinued thereafter. The separate lines connect outcomes on different treatment durations for a given transmission setting. The Ov16 antibody prevalence was estimated assuming that the Ov16 antibody test has a sensitivity of 80%, and specificity of 99%. Note that the horizontal axis is ordered from highest to lowest.

Fig 6 illustrates how patterns would change if Ov16 antibody prevalence would be measured in a wider age group (0–19 years). If antibodies were measured in all those aged <20, the curves relating probability of elimination to Ov16 antibody prevalence becomes less steep and the antibody prevalence threshold below which 95% probability elimination is achieved becomes higher.

**Fig 7:**
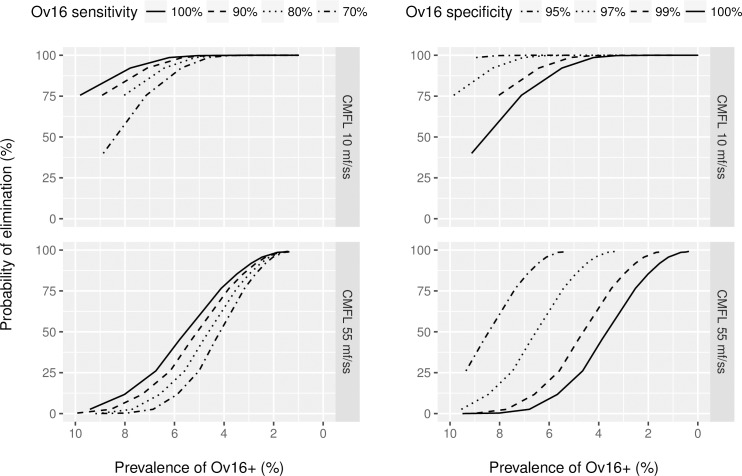
Probability of elimination in relation to the Ov16 antibody prevalence as measured one year after the last treatment, in relation to test characteristics, for scenarios with treatment coverage of 65%. In the figures on the left, sensitivity is varied while specificity is fixed at 99%. In the figures on the right, the sensitivity is fixed at 80%, while specificity is varied. Note that the horizontal axis is ordered from highest to lowest. The lines connect outcomes for different treatment durations with otherwise the same assumptions. The Ov16 antibody prevalence was estimated according to hypothesis 2 in 0–9 year old children.

The observed Ov16 antibody prevalence depends on the sensitivity and specificity of the Ov16 antibody test (Fig 7). A less-sensitive test will under-estimate seroprevalence, and the probability of elimination for a given measured seroprevalence declines with declining test sensitivity. A less-specific test will over-estimate seroprevalence and the probability of elimination for a given measured seroprevalence increases with declining test specificity.

Nota bene, caution is required in the interpretation of Fig 4B and 4C, and similarly of Figs 5–7. These figures show the averages of 1,000 simulations per scenario (minus the number of failed runs). Transmission conditions and treatment history are fixed per scenario, but the resulting post-MDA mf prevalence and Ov16 antibody prevalence vary between runs, as is shown in the figures in S2 Appendix. The presented elimination probabilities are conditional on the underlying variation in prevalence between runs, and runs resulting in elimination may on average have lower post-MDA prevalence than the runs resulting in recrudescence. These results are applicable to groups of communities with similar transmission conditions and history of control. The results should thus not be interpreted as the probability of elimination for a specific site with a given mf or Ov16 antibody prevalence at a particular timepoint.

In this section, we have presented selected findings to highlight the main patterns in our results, concentrating on two settings with either moderate or high transmission intensity and hypothesis 2 only. A complete overview of simulation results for all five transmission settings and the three hypothesized triggers of seroconversion is provided in S3 Appendix.

## Discussion

The ONCHOSIM simulation model was used to explore for different transmission settings how the Ov16 antibody prevalence in children 1 year after the last MDA round is related to the duration and coverage of MDA, the mf prevalence in the population, and the probability that onchocerciasis is eventually eliminated. The post-MDA mf prevalence in the population aged 5 years and above and the Ov16 antibody prevalence in children aged 0–9 years decline with increasing duration and treatment coverage. The association between these infection indicators and the model-predicted probability of elimination was not influenced much by duration and coverage of MDA, but depended strongly on local transmission conditions (for which baseline endemicity is a good proxy). For Ov16, this association further depended on the assumed trigger of seroconversion and diagnostic test characteristics (sensitivity and specificity).

### Age range for seroprevalence assessment

WHO-guidelines for deciding when to stop MDA and verification of elimination after a 3–5 year post-treatment surveillance period, recommend the assessment of Ov16 antibody prevalence in children aged 0–9 in combination with entomological evaluation by O-150 polymerase chain reaction (pool screen) testing in blackflies [12]. Low rates or absent seroconversion in children born after the start of MDA indicates that the force-of-infection has been low during their lifespan. It is important to realize, though, that the post-MDA Ov16 antibody prevalence can also vary within the 0–9 age range, depending on local transmission conditions and the history of MDA (see Fig 3). For accurate interpretation of observed antibody prevalence levels in children, it is therefore critical to know the age distribution among children examined. Somewhat higher seroprevalence levels are expected if infants and toddlers are excluded or underrepresented in the sample, and vice versa. Note that exclusion of the youngest children from the sample will not necessarily cause an important loss of information, since—even in the presence of some ongoing transmission—this age group will mostly be antibody-negative. In any case, age-standardization of sample results may be warranted.

Additional information might be obtained by looking at a wider age range (Fig 6) or by considering the full age-profile (Fig 3). It has previously been shown that broadening the sampled age range may enable discrimination of smaller changes in force of infection in communities approaching elimination [38]. The results in Fig 3 and Fig 6 were derived under the assumption that there is no seroreversion, so that older individuals remain positive and the maximum seroprevalence in the oldest age groups remains unchanged. Under this assumption, the age-span over which Ov16 antibody prevalence is (close to) zero is indicative of the period over which transmission has been low. Yet, the interpretation of seroprevalence data in older age groups is complicated by uncertainty about the possibility and dynamics of seroreversion. The prevalence in all older age groups would be lower than predicted in this study, with the relative difference increasing with increasing duration of MDA. While seroreversion may still be indicative of strongly reduced transmission, it becomes more difficult to estimate the period over which transmission was low.

### Critical threshold for stopping MDA

The ONCHOSIM simulations reveal that local transmission conditions influence the predicted association between the post-MDA mf or Ov16 antibody prevalence levels and probability of elimination. More specifically, mf prevalence and Ov16 antibody prevalence need to be brought down to lower levels in high-transmission settings (with high baseline endemicity) than in low-transmission settings (with low baseline endemicity). This pattern is theoretically expected [39] and in line with predictions in previous onchocerciasis modelling papers [27, 40] and with findings for lymphatic filariasis [41–43].

Readers may be tempted to deduce endpoints for MDA from the results presented in Figs 4–7, but caution is required. Our study was not designed to estimate a critical threshold for the seroprevalence, below which MDA can be interrupted. As explained in the Results section, these figures should not be interpreted as the elimination probability of elimination for a specific community with a given mf or Ov16 antibody prevalence. This requires a different type of analysis that will be presented elsewhere. A more appropriate interpretation is that these figures give the expected elimination probabilities for groups of communities with similar transmission conditions and history of control. This is still not directly applicable to real-life treatment areas, because real-life treatment areas are usually not homogeneous with respect to transmission conditions. Between-community variation in transmission conditions results in strong variation in the expected residual mf prevalence or Ov16 antibody prevalence levels after a given duration of MDA. This distorts the association between the mean residual prevalence and elimination prospects, and complicates the interpretation of mean prevalence levels.

The current guidelines for defining when to stop MDA propose to sample children under 10 for antibody testing by a multistage stratified sampling method scheme applied to the local lower administrative unit level, thereby estimating the mean Ov16 antibody prevalence in a MDA implementation unit [12]. These mean prevalence estimates are subject to the interpretation problems identified above. Low prevalence levels are expected in communities that are relatively far from the breeding sites, while the prevalence may still be above the critical threshold in the communities with highest transmission, i.e. those communities that are closest to the most active fly breeding sites. From an overall mean prevalence, it is difficult to deduce with certainty whether transmission is also interrupted in the high transmission core communities, unless the critical threshold is set at a very low level. It would be more efficient to first assess whether prevalence is brought below the critical threshold in the high-transmission communities closest to the breeding sites; if so, one can proceed with a region-wide assessment to verify that the same is achieved in other communities. Knowledge regarding baseline endemicity level and local transmission conditions in the high-transmission core communities should be considered in defining the critical threshold level to be used in a MDA implementation unit.

### Diagnostic test characteristics

For the purposes of the simulations presented here, a baseline sensitivity of 80% and specificity of 99% were used. An indication of the sensitivity of Ov16 antibody detection test was obtained from Lipner et al, who reported that 76%-81% of mf positives were also Ov16 positive [13]. We implicitly assumed that the sensitivity is independent of age and mf intensity, once an individual has experienced the trigger.

Fig 7 shows how sensitive the association between Ov16 antibody prevalence in children and elimination prospects is to diagnostic test characteristics. It is therefore critical to get better information on these characteristics. It is also important to realize that these test characteristics can differ between different types of test. In all diagnostic tests, but especially in the case of serological tests there is typically a trade-off between sensitivity and specificity defined by the receiver-operating characteristic (ROC) curve of the test. Test characteristics will depend on the platform (ELISA or RDT) and the somewhat arbitrarily-determined threshold concentration of Ov16 IgG4 used to distinguish positive and negative cases. This implies that the association between measured seroprevalence levels and elimination prospects, and hence the endpoint for MDA, may vary from platform to platform (ELISA versus RDT) and within platforms even from assay condition to assay condition. Further standardization of Ov16 antibody testing (e.g. by means of a RDT) is recommended to interpret the measured Ov16 antibody prevalence.

### Uncertainty

In the text above, we have identified several uncertain factors that can influence our predictions and endpoints for MDA, including uncertainties regarding the trigger and dynamics of the IgG4 immune responsiveness against Ov16 antigen and diagnostic test characteristics. We considered three different hypotheses with respect to the trigger of seroconversion. Our modelling shows that Ov16 antibody prevalence is relatively non-informative for predicting elimination if seroconversion is associated with mf production (hypothesis 3). The disparity between hypothesis 1 and 2 on the one hand and hypothesis 3 on the other in the association between antibody prevalence levels in children and elimination probability is large (see Fig 4), which would lead to very different estimates of the critical threshold for the antibody prevalence in children. We further assumed that: seroconversion occurs immediately after experiencing the trigger, a fixed proportion of individuals will never become seropositive even after experiencing the trigger, no seroreversion occurs, and diagnostic test characteristics are independent of an individual’s age and worm load. The reality may be more complex, which could influence the association between post-MDA prevalence levels and likelihood of elimination and the predictive value of antibody tests. Our conclusions in qualitative terms may not be influenced much by the identified uncertainties, but any quantitative predictions should be interpreted with care until better information is available regarding the trigger and dynamics of antibody responsiveness.

Better understanding is to come from empirical data. For example, pre-control empirical data on Ov16 serostatus and mf status by age may help to elucidate the trigger and dynamics of seroconversion. We analyzed such data from two villages in Côte d’Ivoire that were highly endemic for onchocerciasis and had no history of control (Mafia: study population n = 134, mf prevalence 83%; CMFL 8.6 mf/snip; Zakpaberi: study population n = 367, mf prevalence 65%, CMFL 7.4 mf/snip) [13] (individual level data were kindly provided by the authors). These data showed that in children mf and Ov16 antibody prevalence levels are about the same (out of 20 children <10, 11 (55%) and 10 (50%) were mf and antibody positive in Mafia, respectively; similarly, out of 124 children <10, 48 (39%) and 56 (45%) were mf and antibody positive in Zakpaberi). In adults, the mf prevalence was almost always higher than the Ov16 antibody prevalence. Firm conclusions regarding the trigger of seroconversion and antibody test sensitivity (as defined in the model) could not be drawn: the model could reproduce age patterns of mf and Ov16 seropositivity with each of the three hypothesized triggers, although we had to assume a lower sensitivity of the serological test if seroconversion was triggered by the establishment of the first worms (hypothesis 1 or 2) than if it was triggered by mf production (hypothesis 3). Additional pre-control data may become available from recent (pre-control) mapping studies, with information on both mf counts and Ov16 antibody test results. Better information on diagnostic test characteristics (sensitivity, specificity and positive and negative predictive values) is to come from diagnostic test comparison studies. Post-MDA data from epidemiological evaluation studies (e.g. [44–47, 38]) can also be useful, although the informativeness of such data depends on the quality of information on local transmission dynamics and the history of control.

### Conclusions

This work provides valuable insight into the factors that influence post-MDA seroprevalence levels and age patterns of Ov16 seropositivity. The post-treatment antibody prevalence in children was found to be a good indicator of probability of elimination, although the association is dependent on local transmission conditions and assumptions regarding uncertain factors such as the trigger and dynamics of IgG4 responsiveness and diagnostic test characteristics. These uncertainties hinder the estimation of a critical threshold that can be taken as endpoint for MDA programmes and better information should come from empirical data. Yet, our study clearly demonstrates that this threshold will be dependent on baseline endemicity levels, which should be taken into account in guidelines for defining when to stop MDA.

## Supporting Information

S1 Appendix Model input specifications: probability distributions, functions and parameter values. (PDF)

S2 Appendix Stochastic variants of Figs 1 and 2, showing the variability in predicted mf prevalence for 100 runs. (PDF)

S3 Appendix Supplementary figures presenting the results for all simulated transmission settings. (PDF)
