# Supplementary material for: Modelling Anti-Ov16 IgG4 Antibody Prevalence as an Indicator for Evaluation and Decision Making in Onchocerciasis Elimination Programmes
Source: PLoS Negl Trop Dis. 2017 Jan 23;11(1):e0005314. doi: 10.1371/journal.pntd.0005314 (PMC5289624; doi:10.1371/journal.pntd.0005314)
Supplement: S1 Appendix — (PDF) [file pntd.0005314.s001.pdf]

S1 Appendix
Model input specifications: probability distributions, functions and parameter values

1 WORMSIM version

A detailed formal description of the ONCHOSIM model with JAVA program code is provided elsewhere for version 2.58Ap9 of the model (see additional files 1 and 2 in [1]). For the current study, ONCHOSIM version 2.74 was used, which incorporates extra output concerning the Ov16 serostatus of individuals based on their history of infection, as described below. Some other refinements were made in this model version, and these are highlighted in the annotated input file below. They are not discussed in detail because they are not relevant for the work presented here.

2 Annotated input file

The WORMSIM inputfile is an XML file that can be edited with any text editor or alternatively, with an XML editor (such as Oxygen XML Editor). The advantage of using the XML format is that any input file can be validated against an XML Schema (a formal specification of the grammar used in the specific XML dialect used for the WORMSIM input file).

A copy of an annotated input file is included below, showing the input assumptions as used in this study to simulate onchocerciasis transmission with WORMSIM version 2.74. The documentation is split into fragments that cover the different elements of the input file (gray-shaded boxes). Together, these fragments constitute a complete input file. The following elements are distinguished:

- Inputfile header
- Simulation
- Demography
- Blindness
- Exposure
- Immunity
- Worm
- Fly
- Mass.treatment
- Vector.control

Meaning of the formatting of the input files: Text formatted in green as `<!–- this is a comment -->` denotes a comment. Grouping name tags for sets of input parameters are displayed in **blue**, while **red** indicates the specific parameters for which input is to be given. The actual inputs are found in the quotation marks, formatted in **purple**.

2.1 Input file header

```
<?xml version="1.0" encoding="UTF-8"?>
<wormsim.inputfile xmlns:xsi="http://www.w3.org/2001/XMLSchema-instance"
                xsi:noNamespaceSchemaLocation="wormsim.xsd" >
<!-- Input file for WORMSIM: -->
<!-- Wormsim v2.74 -->
<!-- Author: Yvonne Lont, Wilma Stolk -->
```

2.2 Simulation

The <simulation> element specifies the timing of surveys (i.e. output moments), the number of skin snips taken at each survey and the age classes for output and the duration of the warming-up period (also called burn=in period). A simulation always starts with an uninfected human population. To trigger the transmission, one can introduce parasites in the initial population by letting an intial force-of-infection act upon it during a given period preceding the actual simulation (see subsection on exposure for further information on the initial force-of-infection). The actual simulation starts with a long warming-up period (200 years), which is used to make sure that both the human population composition and the parasite population are in a dynamic equilibrium before the start of the surveillance and introduction of nterventions. The level of the dynamic equilibrium depends on the assumptions regarding the transmission dynamics and chance effects. The warming-up period does not always result in a stable endemic situation. Especially when biting rates are low, the infection may by chance go extinct.

```
<!-- general settings for simulation and simulation output       -->
<simulation>
       <!-- number of skin snip taken per person                  -->
       <!-- and whether output should be provided at              -->
       <!-- individual level (true) or not(“false”)               -->
       <surveillance nr.skin-snips="2" individual-output = "false">
              <!-- timing of surveys                               -->
              <!-- month 0 represents January 1st                  -->
              <!-- see note regarding "delay" below                -->
              <start year="1990" month="6" delay="-2"/>
              <stop year="2069" month="7"/>
              <interval years="1" months="0"/>
              <!-- upper bounds of age categories in output      -->
              <age.classes>
                     <age.class age.limit="1"/>
                     <age.class age.limit="2"/>
                     <age.class age.limit="3"/>
                     <age.class age.limit="4"/>
                     <age.class age.limit="5"/>
                     <age.class age.limit="6"/>
                     <age.class age.limit="7"/>
                     <age.class age.limit="8"/>
                     <age.class age.limit="9"/>
                     <age.class age.limit="10"/>
                     <age.class age.limit="11"/>
                     <age.class age.limit="12"/>
                     <age.class age.limit="13"/>
                     <age.class age.limit="14"/>
                     <age.class age.limit="15"/>
                     <age.class age.limit="20"/>
                     <age.class age.limit="25"/>
                     <age.class age.limit="30"/>
                     <age.class age.limit="35"/>
                     <age.class age.limit="40"/>
                     <age.class age.limit="50"/>
                     <age.class age.limit="90"/>
              </age.classes>
       </surveillance>
       <warming.up duration="200"/>
       <!-- upper bounds and weights of reference population for -->
       <!-- age and sex-standardized output (OCP standard pop)    -->
       <!-- Standardization is not used for this study            -->
       <standard.population>
              <age.group age.limit="5" n.males="1401" n.females="1353"/>
              <age.group age.limit="10" n.males="1769" n.females="1507"/>
              <age.group age.limit="15" n.males="1739" n.females="1465"/>
              <age.group age.limit="20" n.males="1085" n.females="921"/>
              <age.group age.limit="30" n.males="1409" n.females="1738"/>
              <age.group age.limit="50" n.males="2388" n.females="2821"/>
              <age.group age.limit="90" n.males="1208" n.females="1237"/>
       </standard.population>
</simulation>
```

2.3 Demography

The <demography> element defines life tables for the male and female population, the maximum population size (above which random persons will be removed), a fertility table, and the initial population size and age distribution. See comments below.

```
<!-- demographic parameters of simulated population              -->
<demography>
       <!-- whenever the simulated population size exceeds the   -->
       <!-- the specified maximum, a random fraction is removed  -->
       <!-- see note regarding "delay" below                     -->
       <the.reaper max.population.size="440" reap="0.1" delay="-3"/>
       <!-- survival represents cumulative survival probability  -->
       <!-- and is determined by for unspecified ages by linear  -->
       <!-- interpolation of values for specified age limits     -->
       <life.table>
              <survival age.limit="5" male.survival="0.804" female.survival="0.804"/>
              <survival age.limit="10" male.survival="0.772" female.survival="0.772"/>
              <survival age.limit="15" male.survival="0.76" female.survival="0.76"/>
              <survival age.limit="20" male.survival="0.74" female.survival="0.74"/>
              <survival age.limit="30" male.survival="0.686" female.survival="0.686"/>
              <survival age.limit="50" male.survival="0.509" female.survival="0.509"/>
              <survival age.limit="90" male.survival="0" female.survival="0"/>
       </life.table>
       <!-- rates represent probabilies for women to give birth  -->
       <!-- to one child in some year, given a woman's age    -->
       <!-- rates are assumed constant within each age category  -->
       <!-- and ages limits represent upper bounds of categories -->
       <!-- see note regarding "delay" below                       -->
       <fertility.table delay="-4">
              <fertility age.limit="5" birth.rate="0"/>
              <fertility age.limit="10" birth.rate="0"/>
              <fertility age.limit="15" birth.rate="0"/>
              <fertility age.limit="20" birth.rate="0.109"/>
              <fertility age.limit="30" birth.rate="0.3"/>
              <fertility age.limit="50" birth.rate="0.119"/>
              <fertility age.limit="90" birth.rate="0"/>
       </fertility.table>
       <!-- population size to start simulation with                   -->
       <initial.population>
              <age.group age.limit="5" n.males="4" n.females="4"/>
              <age.group age.limit="10" n.males="5" n.females="5"/>
              <age.group age.limit="15" n.males="3" n.females="3"/>
              <age.group age.limit="20" n.males="3" n.females="3"/>
              <age.group age.limit="30" n.males="4" n.females="4"/>
              <age.group age.limit="50" n.males="6" n.females="6"/>
              <age.group age.limit="90" n.males="4" n.females="4"/>
       </initial.population>
</demography>
```

2.4 Morbidity

The <morbidity> element defines the parameters for development of morbidity, in this case "visual impairment" and "blindness". This morbidity module is refined version of the orginal morbidity module in ONCHOSIM as described in our earlier publications. As in the original version, morbidity is assumed to result from damage induced by mf that accumulates over time. The refined model allows to simulate different sequential stages of this disease (such as visual impairment which can later progress into blindness) and allows for regression of mf-induced damage. Reaching a certain disease stage can lead to a reduction in remaining life expectancy. The parameter values were chosen in such a way, that model-predicted patterns of morbidity by age and sex are similar to those predicted with the original model.

```
<!-- parameters for development of visual impairment and blindness -->
<!-- when a person's cumulative exposure to mf exceeds the first   -->
<!-- and second threshold, respectively, a person is considered    -->
<!-- to be visually impaired or blind. Individual variation in     -->
<!-- individual variation in susceptibility is modeled by letting  -->
<!-- ting the progression rate vary between individuals, assuming  -->
<!—a Weibull distribution with a user-specified shape parameter    -->
```

```
<disease.processes>
	<disease.process name="damage-due-to-mf" susceptibility.shape.param="2">
		<regression.rate fun.nr="0" a="0"/>
		<disease.stage name="stage-zero" treshold="0"/>
		<disease.stage name="stage-one" treshold="4000">
			<symptom name="VI"/>
		</disease.stage>
		<disease.stage name="stage-two" treshold="7000">
			<symptom name="blind"/>
		</disease.stage>
	</disease.process>
</disease.processes>
<!-- Upon turning blind, the life-expectancy of a person -->
<!-- is reduced by a variable fraction: on average 50%,  -->
<!-- and uniformly distributed between 0% and 100%.      -->
<!-- VI does not influence the life expectancy.          -->
<symptom.defs>
	<symptom.def name="VI">
		<pct-life-expectancy-reduction dist.nr="0" mean="0"/>
	</symptom.def>
	<symptom.def name="blind" irreversible="true">
		<pct-life-expectancy-reduction dist.nr="1" min="0" max="100" mean="50"/>
	</symptom.def>
</symptom.defs>
```

2.5 Exposure

The <exposure> element defines the parameters for the exposure of humans to a vector and thereby the contribution of humans to the vector cloud. WORMSIM version 2.58Ap9 allows for different exposure and contribution function, which is relevant when modelling soil-transmitted helminthiasis; this distinction is not relevant for onchocerciasis and version 2.74 does not distinguish between exposure and contribution functions.

```
<!-- parameters for exposure to fly bites                     -->
<!-- N.B. in WORMSIM we only describe fly bites on humans,    -->
<exposure>
	<!-- initial force of infection to introduce infection  -->
	<!-- into the simulated population at the start of the  -->
	<!-- warming up period; duration in years               -->
	<initial.foi duration="7.5" foi="4"/>
	<!-- parameters for individual exposure to fly bites,   -->
	<!-- depending on gender, age, and personal factors     -->
	<male>
		<!-- age-dependent exposure, relative to mean exp -->
		<!-- of adult males, assuming a linear increase   -->
		<!-- between age 0 and 20, after which exposure   -->
		<!-- is 1.0                                       -->
		<exposure.function fun.nr="1" a="0.05" c="1"/>
		<!-- individual variation in exposure related to  -->
		<!-- e.g. occupation and attractiveness to flies, -->
		<!-- assuming a gamma distribution with mean one  -->
		<!-- and variation 1/p1 (shape and rate p1),      -->
		<!-- truncated by "min" and "max"                 -->
		<exposure.index dist.nr="4" min="0" max="20" p1="3.5"/>
	</male>
	<female>
		<!-- age-dependent exposure, relative to mean exp -->
		<!-- of adult males, assuming a linear increase   -->
		<!-- between age 0 and 20, after which exposure   -->
		<!-- is 0.70 of the level in males                -->
		<exposure.function fun.nr="1" a="0.035" c="0.7"/>
		<!-- individual variation in exposure related to  -->
		<!-- e.g. occupation and attractiveness to flies, -->
		<!-- assuming a gamma distribution with mean one  -->
		<!-- and variation 1/p1 (shape and rate p1),      -->
		<!-- truncated by "min" and "max"                 -->
		<exposure.index dist.nr="4" min="0" max="20" p1="3.5"/>
	</female>
</exposure>
```

2.6 Immunity

The <immunity> element defines the (optional) development of host immunity. Immunity is not considered in the current model.

```
<!-- parameters related to development of host immunity         -->
<!-- these are currently set such that no immunity develops     -->
<immunity>
        <male alpha="0" beta="1">
                <immunity.function fun.nr="0" a="1"/>
                <immunity.index dist.nr="0" min="0" max="20"/>
        </male>
        <female alpha="0" beta="1">
                <immunity.function fun.nr="0" a="1"/>
                <immunity.index dist.nr="0" min="0" max="20"/>
        </female>
</immunity>
```

2.7 Worm

The <worm> element defines parameters for worm lifespan, prepatent period, mating between M and F worms, age-dependent production of microfilaria, mf density per worm and skin dispersal.

```
<!-- parameters for worm survival and mf production             -->
<!-- mf lifespan in months, see note regarding "delay" below    -->
<worm mf-lifespan="9" monthly.event.delay="1">
        <!-- worm lifespan in years, allowing for variation      -->
        <!-- between worms, assuming a Weibull distribution with -->
        <!-- mean 10 and shape 3.76, bounded by "min" and "max"  -->
        <lifespan dist.nr="3" min="0" max="50" mean="10" p1="3.76"/>
        <!-- pre-patent during which worms do not produce mf and -->
        <!-- are not affected by ivermectin                      -->
        <prepatent.period dist.nr="0" mean="1"/>
        <!-- number of months a female can produce mf with one   -->
        <!-- insemination, and number of females one male worm   -->
        <!-- can inseminate per month                            -->
        <!-- if there are more female worms than the total male  -->
        <!-- potential, every female has a probability of being  -->
        <!-- inseminated equal to N_mw/N_fm*male.potential       -->
        <mating cycle="3" male.potential="100"/>
        <!-- mf production by female worms as function of worm   -->
        <!-- age minus pre-patent period; mf production at un-   -->
        <!-- specified ages is determined by linear interpol.    -->
        <age.dependent.mf-production>
                <mf-production age.limit="0" production="1"/>
                <mf-production age.limit="5" production="1"/>
                <mf-production age.limit="20" production="0"/>
        </age.dependent.mf-production>
        <!-- expected N_mf per worm in skin snip as product of  -->
        <!-- number of mf contributed per fully fecund worm and -->
        <!-- random dispersal factor representing the distance  -->
        <!-- between a worm and site of skin snip, assuming an  -->
        <!-- exponential distribution, truncated by "min" and   -->
        <!-- "max"                                              -->
        <skin.mf-density.per.worm fun.nr="1" a="7.6" c="-1"/>
        <skin.dispersal dist.nr="2" min="0" max="5"/>
        <!-- poisson distribution for observed number of mf in  -->
        <!-- one skin snip                                      -->
        <skin-snip.variability dist.nr="5"/>
</worm>
```

2.8 Fly

The <fly> element defines parameters that determine the successful uptake and development of L1 larvae into infective L3 larvae and also determins the fly biting rate.

```
<!-- probability that an mf taken up by a fly bite develops    -->
<!-- into an L3 and is transmitted to another human (taking    -->
<!-- account of the fly's gonotropic cycle, survival, and      -->
<!-- duration and probability of an ingested mf developing     -->
<!-- into an infective L3 and surviving up to the point of     -->
```

```
        <!-- transmission                                              -->
        <fly transmission.probability="0.07345">
                <!-- functional relation between uptake of mf and mf    -->
                <!-- density in the skin, assuming exponential satu-    -->
                <!-- ration to maximum level a with initial slope b     -->
                <!-- and shape c                                        -->
                <L1-uptake fun.nr="3" a="1.2" b="0.0213" c="0.0861"/>
                <!-- seasonal pattern in monthly biting rates (mbr),    -->
                <!-- as observed in Asubende, Ghana                    -->
                <!-- in the simulation, actual biting rates for an      -->
                <!-- individual are calculated as product of monthly    -->
                <!-- biting rate in Asubende, a factor representing     -->
                <!-- the mean exposure in adult males in the simulated  -->
                <!-- village relative to Asubende ("relative biting     -->
                <!-- rate"), and all other factors related to gender,   -->
                <!-- age, and individual variation in exposure         -->
                <!-- to produce some desired endemicity level in the    -->
                <!-- simulation, adjust the relative biting rate such   -->
                <!-- that mf prevalence or density (distribution) in    -->
                <!-- the population (output at the desired time point)  -->
                <!-- equals the desired value                          -->
                <!-- N.B. individual variation in exposure to fly bites -->
                <!-- also determined mean and distribution of simulated -->
                <!-- infection levels                                  -->
                <!-- rbr = 0.305 for CMFL 5-->
                <!-- rbr = 0.329 for CMFL 10-->
                <!-- rbr = 0.457 for CMFL 30-->
                <!-- rbr = 0.586 for CMFL 55-->
                <!-- rbr = 0.720 for CMFL 80-->
                <monthly.biting.rates relative.biting.rate="0.305">
                        <mbr month="1"  rate="2670"/>
                        <mbr month="2"  rate="2350"/>
                        <mbr month="3"  rate="1500"/>
                        <mbr month="4"  rate="1920"/>
                        <mbr month="5"  rate="1940"/>
                        <mbr month="6"  rate="1690"/>
                        <mbr month="7"  rate="2630"/>
                        <mbr month="8"  rate="3410"/>
                        <mbr month="9"  rate="3010"/>
                        <mbr month="10" rate="3290"/>
                        <mbr month="11" rate="3750"/>
                        <mbr month="12" rate="2690"/>
                </monthly.biting.rates>
        </fly>
```

2.9 Mass treatment

The <mass.treatment> element defines parameters for the timing of mass treatment rounds, individual compliance (permanent, temporary and age dependent), and effects of employed drugs on mature worms, mf production by F worms and on mf. The mass treatment module in version 2.74 allows for different drugs to be used in mass treatment and different efficacy mechanisms. The statement v58="true" indicates that the mechanisms employed here is the same as in the previously published WORMSIM variant 2.58Ap9, and corresponding model-parameters are given under v58.drugs.

```
<!-- parameters for mass treatment                             -->
<!-- The statement v58="true" indicates that the mechanisms    -->
        <!-- employed here is the same as in the previously published  -->
        <!-- WORMSIM variant 2.58Ap9-->
<mass.treatment v58="true">
        <compliance.options>
                <!-- random fraction of population permanently not      -->
                <!-- eligible for treatment due to chronic illness and  -->
                <!-- random fraction of population in which ivermectin  -->
                <!-- does not work due to diarrhoe (temporary effect)   -->
                <compliance name="default" fraction.excluded="0.025"
fraction.malabsorption="0.025" compliance.model="0">
                        <!-- weights for age and sex-specific compliance, given -->
                        <!-- some expected overall coverage in the eligible     -->
                        <!-- population; weights are constant within age groups -->
                        <age.and.sex.specific.compliance age.limit="5" male.compliance="0"
female.compliance="0"/>
```

```
                        <age.and.sex.specific.compliance age.limit="10" male.compliance="0.75"
female.compliance="0.75"/>
                        <age.and.sex.specific.compliance age.limit="15" male.compliance="0.8"
female.compliance="0.7"/>
                        <age.and.sex.specific.compliance age.limit="20" male.compliance="0.8"
female.compliance="0.74"/>
                        <age.and.sex.specific.compliance age.limit="30" male.compliance="0.7"
female.compliance="0.65"/>
                        <age.and.sex.specific.compliance age.limit="50" male.compliance="0.75"
female.compliance="0.7"/>
                        <age.and.sex.specific.compliance age.limit="90" male.compliance="0.8"
female.compliance="0.75"/>
                </compliance>
        </compliance.options>
        <treatment.rounds>
                <!-- Timing of individual mass treatment rounds (one   -->
                <!-- line per mass treatment round), specifying year,  -->
                <!-- month (0 represents January 1st), and population  -->
                <!-- coverage (fraction of total village population,   -->
                <!-- including those not eligible for treatment).      -->
                <!-- Varying between simulated scenarios               -->
                <!-- See note regarding "delay" below                  -->
                <treatment.round year="1995" month="6" drug="ivm" coverage="0.5" delay="-1"/>
        </treatment.rounds>
                <v58.drugs>
                        <!-- ivermectin efficacy, specified according to mechanisms 2.58, -->
                        <!-- as permanent reduction in worm capacity to produce mf        -->
                        <!-- (cumulative effects allowed), pattern of how mf production   -->
                        <!-- recovers over time (to a new, reduced maximum level)         -->
                        <!-- and fraction of mf surviving each treatment                  -->
                <v58.drug name="ivm" compliance="default" include.prepatent.worms="true">
                        <v58.treatment.effects permanent.reduction.mf-production="0.349"
                                period.of.recovery="0.875"
shape.parameter.recovery.function="1.483"
                                fraction.killed="0">
                                <fraction.mf.surviving dist.nr="0" mean="0.0"/>
                                <!-- variability in treatment effects (relative to mean,    -->
                                <!-- expected effect), assuming a Weibull distribution with -->
                                <!-- mean one and shape "p1"                                -->
                                <treatment.effect.variability dist.nr="3" mean="1.0" p1="2"/>
                        </v58.treatment.effects>
                </v58.drug>
        </v58.drugs>
</mass.treatment>
```

2.10 Vector control

The <vector.control> element defines parameters for setting the effectivity of vector control during periods of vector control.

```
<!—Vector control is not used in this study, and the element can stay empty   -->
        <vector.control>
        </vector.control>
</wormsim.inputfile>

<!-- In ONCHOSIM, some events may be scheduled at the same time. The attribute     -->
<!-- "delay" specifies at what time an event takes place, relative to other events -->
<!-- planned at the same time. The order of events is currently: human births, the -->
<!-- reaper, survey, mass treatment, worm and mf generation and death.             -->
```

3 Summary table of input specifications

Parameter	Value	Source
**Human demography**		
Cumulative survival ($F(a)$), by age (assumed to be the same for males and females)		[2]
0	1.000	
5	0.804	
10	0.772	
15	0.760	
20	0.740	
30	0.686	
50	0.509	
90	0.000	
Fertility rate per woman ($R(a)$), by age		[2]
0-1	0.000	
15-19	0.109	
20-29	0.300	
30-49	0.119	
50+	0.000	
Population trimming	10% if population size exceeds 440.	Assumption
**Transmission of infection**[a]		
General transmission parameters		
Relative biting rate (*rbr*)	Varied between simulations to modify the annual biting rate.	
Seasonal variation in contribution to reservoir (*mbr*)	104%, 91%, 58%, 75%, 75%, 66%, 102%, 133%, 117%, 128%, 146%, and 105% times the average monthly biting rate (January–December)	[3]
Transmission probability (*v*), i.e. the probability that an infective particle in the reservoir successfully develops into a parasite life stage that is capable of infecting a human host	$v = 0.07345$; see reference for the derivation of this value, given parameters for fly biology and development of infective L3 larvae within the fly.	[4]
Individual relative exposure to flies		
Relative exposure by age and sex (*Exa*)	Zero at birth, linearly increasing between ages 0–20 from 0 to 1.0 for men and from 0 to 0.7 for women, and then constant from the age of 20 years onwards	[5]

[a] Some previous versions of ONCHOSIM required input on success ratio (sr) and zoophily rate (z). In version 2.74 they are "hard" embedded in the computer code and not modifiable, but the values are the same as reported elsewhere: sr = 0.31% {Plaisier, 1996 #1358; Duke, 1993 #2932} and zoophily = 0.04 {Habbema, 1996 #2912}, expert opinion (OCP entomologists).

Parameter	Value	Source
Variation due to personal factors (fixed through life) given age and sex (α_{Exi})	Gamma distribution with mean 1.0 and shape and rate equal to 3.5	[5], unpublished data from OCP
Host immunity to incoming infections		
Average impact of host immunity (α_{Imm})	Assumed irrelevant for onchocerciasis, hence $\alpha_{Imm} = 0$; i.e. no effect of immunity on incoming infections.	Assumption
Immunological memory (β_{Imm})	Irrelevant given that $\alpha_{Imm} = 0$.	Assumption
**Life history and productivity of the parasite in the human host**		
Average worm lifespan (*Tl*)	10 years	[6]
Variation in worm lifespan	Weibull distribution with shape 3.76	Assumption [6]
Prepatent period (*pp*)	1 year	[6], which refers to [7, 8]
Age-dependent microfilaria production capacity (*R(a)*)	*R(a)* = 1 for 0 ≤ *a* < 5<br>*R(a)* = 1-((a-5)/15) for 5 ≤ *a* < 20<br>*R(a)* = 0 for *a* > 20	[6], which refers to [9, 10]
Longevity of microfilariae within host (*Tm*)	9 months	[5]
Mating cycle (*rc*)	3 months	[5], which refers to [11, 12]
Male potential (*pot*)	100 female worms.	[5]
Density-dependent female worm reproductive capacity		
Per-worm contribution to host load of infective material ($O(.)$)	7.6 mf/worm	[5]
Exponential saturation of individual female worm productivity per worm present in host (λ_z)	$\lambda_z = 0$, i.e. no exponential saturation.	Assumption
**Morbidity**		
Susceptibility shape parameter	2	Assumption
Regression rate	No regression (constant 0)	Assumption
Disease thresholds	For stage 1, visual impairment: 4000<br>For stage 2, blindness: 7000	Chosen to reproduce morbidity patterns of [13]

Parameter	Value	Source
Reduction in remaining life expectancy due to morbidity	For stage 1, visual impairment: 0%<br>For stage 2, blindness: 50%	[13], which refers to partly published data from OCP [14]; and [15], which refers to [16, 17]
**Infection dynamics in the vector cloud**		
Uptake of infectious material by vector (uptake curve $U(.)$)	Exponential saturating function<br>$f(x) = a(1 - e^{-bx})(1 + e^{-cx})$<br>with parameters *a* = 1.2, *b* = 0.0213, and *c* = 0.0861.	[18], which refers to [19, 20]
Monthly cumulative survival of infective material in the central reservoir (ψ)	0%; i.e. the cloud represents a cloud of vectors that transmit infection within the same month.	Assumption
**Mass treatment coverage**		
Coverage (C_w)	Varied between simulations as explained in text	
Proportion of individuals who never participate in mass treatment	2.5%	Assumption
Proportion of treated people in whom treatment is completely ineffective (randomly selected from all treated)	2.5%	Assumption
Relative compliance ($c_r(k,s)$) by age and sex (descriptive label used in graphs)		Based on unpublished OCP data

age-group	cr(k,males)	cr(k,females)
0-4	0	0
5-9	0.75	0.75
10-14	0.8	0.7
15-19	0.8	0.74
20-29	0.7	0.65
30-49	0.75	0.7
50+	0.8	0.75

Parameter	Value	Source
**Drug treatment**		
Proportion of microfilariae cleared from host	100%	[21]
Duration of temporary reduction in female reproductive capacity (Tr_0), average	11 months	[21]
Permanent reduction in female worm reproductive capacity (d_0), average	34.9%	[21]
Proportion of adult worms killed (m_0)	0%	[21]
Relative effectiveness (v)	Weibull distribution with mean 1 and shape 2	[21]
**Vector control**		
Timing	Not used.	

Parameter	Value	Source
Coverage	Not used.	
**Surveys and diagnosis**		
Nr. of skin snips taken in surveys	2	Assumption
Dispersal factor for worm contribution to measured density of infective material (*d*)	Exponential distribution with mean 1	[6]
Variability in measured host load of infective material (eggs per gram faeces)	Poisson distribution with mean $ss(t)$	[6]

References

1. Stolk, W.A., et al., *Required duration of mass ivermectin treatment for onchocerciasis elimination in Africa: a comparative modelling analysis.* Parasit Vectors, 2015. **8**(1): p. 552.
2. United Nations Department of Economic and Social Affairs Population Division, *World Population Prospects: the 2012 revision, Volume I: Comprehensive Tables.* 2013.
3. Alley, E.S., et al., *The impact of five years of annual ivermectin treatment on skin microfilarial loads in the onchocerciasis focus of Asubende, Ghana.* Trans R Soc Trop Med Hyg, 1994. **88**(5): p. 581-4.
4. Coffeng, L.E., et al., *Elimination of African onchocerciasis: modeling the impact of increasing the frequency of ivermectin mass treatment.* PLoS One, 2014. **9**(12): p. e115886.
5. Plaisier, A.P., *Modelling onchocerciasis transmission and control*, in *Department of Public Health*. 1996, Erasmus University Rotterdam: Rotterdam, the Netherlands. p. 181.
6. Plaisier, A.P., et al., *The reproductive lifespan of Onchocerca volvulus in West African savanna.* Acta Trop, 1991. **48**(4): p. 271-84.
7. Duke, B.O., *Observations on Onchocerca volvulus in experimentally infected chimpanzees.* Tropenmed Parasitol, 1980. **31**(1): p. 41-54.
8. Prost, A., *[Latency period in onchocerciasis].* Bull World Health Organ, 1980. **58**(6): p. 923-5.
9. Albiez, E.J., *Calcification in adult Onchocerca volvulus.* Trop Med Parasitol, 1985. **36**(3): p. 180-1.
10. Karam, M., H. Schulz-Key, and J. Remme, *Population dynamics of Onchocerca volvulus after 7 to 8 years of vector control in West Africa.* Acta Trop, 1987. **44**(4): p. 445-57.
11. Schulz-Key, H. and M. Karam, *Periodic reproduction of Onchocerca volvulus.* Parasitol Today, 1986. **2**(10): p. 284-6.
12. Schulz-Key, H., *Observations on the reproductive biology of Onchocerca volvulus.* Acta Leiden, 1990. **59**(1-2): p. 27-44.
13. Coffeng, L.E., et al., *African Programme For Onchocerciasis Control 1995-2015: model-estimated health impact and cost.* PLoS Negl Trop Dis, 2013. **7**(1): p. e2032.
14. Dadzie, K.Y., et al., *The effect of 7-8 years of vector control on the evolution of ocular onchocerciasis in West African savanna.* Trop Med Parasitol, 1986. **37**: p. 263-70.
15. Plaisier, A.P., et al., *ONCHOSIM: a model and computer simulation program for the transmission and control of onchocerciasis.* Comput Methods Programs Biomed, 1990. **31**(1): p. 43-56.
16. Prost, A. and J. Vaugelade, *[Excess mortality among blind persons in the West African savannah zone].* Bull World Health Organ, 1981. **59**(5): p. 773-6.
17. Kirkwood, B., et al., *Relationships between mortality, visual acuity and microfilarial load in the area of the Onchocerciasis Control Programme.* Trans R Soc Trop Med Hyg, 1983. **77**(6): p. 862-8.
18. Plaisier, A.P., et al., *The risk and dynamics of onchocerciasis recrudescence after cessation of vector control.* Bull World Health Organ, 1991. **69**(2): p. 169-78.
19. World Health Organization, *Onchocerciasis Control Programme in West Africa: report of the annual OCP research meeting, 20-24 March 1989.* (unpublished), 1989.
20. Phillipon, B., *Étude de la transmission d'Onchocerca volvulus (Leuckart, 1893) (Nematoda, Onchocercidae) par Simulium damnosum Theobald, 1903 (Diptera, Simuliidae) en Afrique tropicale. .* 1977, O.R.S.T.O.M.: Paris.
21. Plaisier, A.P., et al., *Irreversible effects of ivermectin on adult parasites in onchocerciasis patients in the Onchocerciasis Control Programme in West Africa.* J Infect Dis, 1995. **172**(1): p. 204-10.
