# Supplementary material for: Modelling Anti-Ov16 IgG4 Antibody Prevalence as an Indicator for Evaluation and Decision Making in Onchocerciasis Elimination Programmes
Source: PLoS Negl Trop Dis. 2017 Jan 23;11(1):e0005314. doi: 10.1371/journal.pntd.0005314 (PMC5289624; doi:10.1371/journal.pntd.0005314)
Supplement: S2 Appendix — (PDF) [file pntd.0005314.s002.pdf]

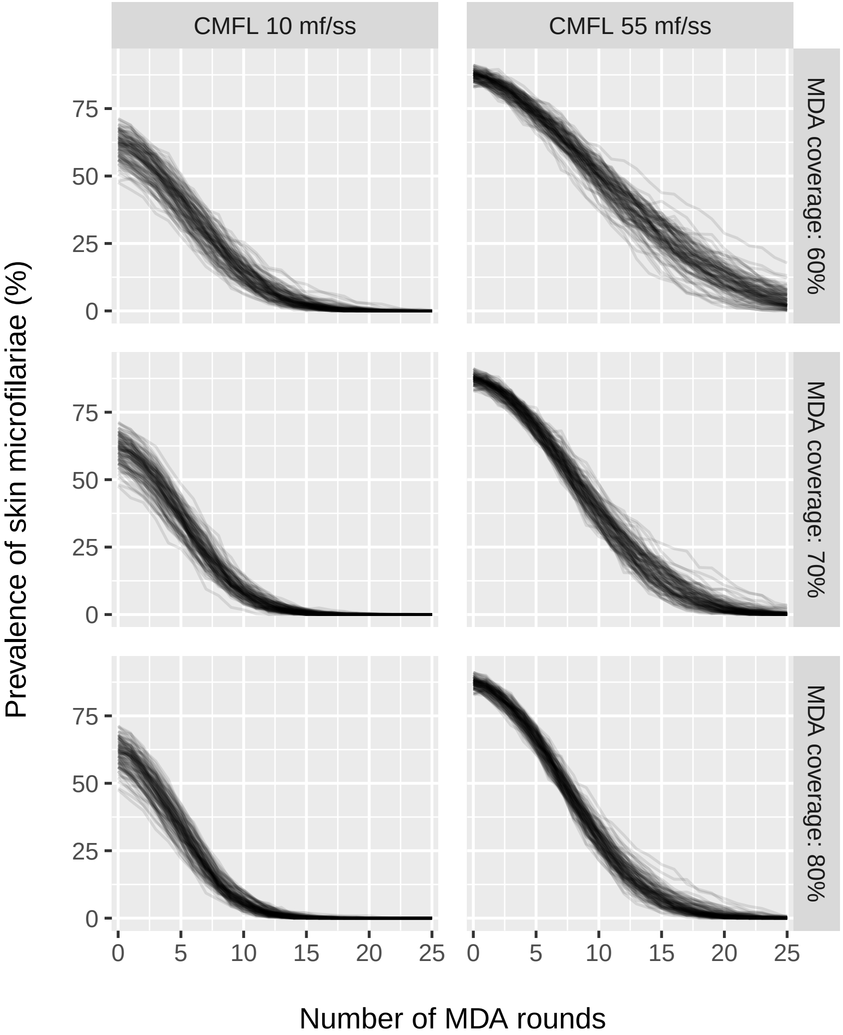
CMFL 10 mf/ss
CMFL 55 mf/ss
MDA coverage: 60%
MDA coverage: 70%
MDA coverage: 80%
Prevalence of skin microfilariae (%)
Number of MDA rounds
0
25
50
75
0
5
10
15
20
25

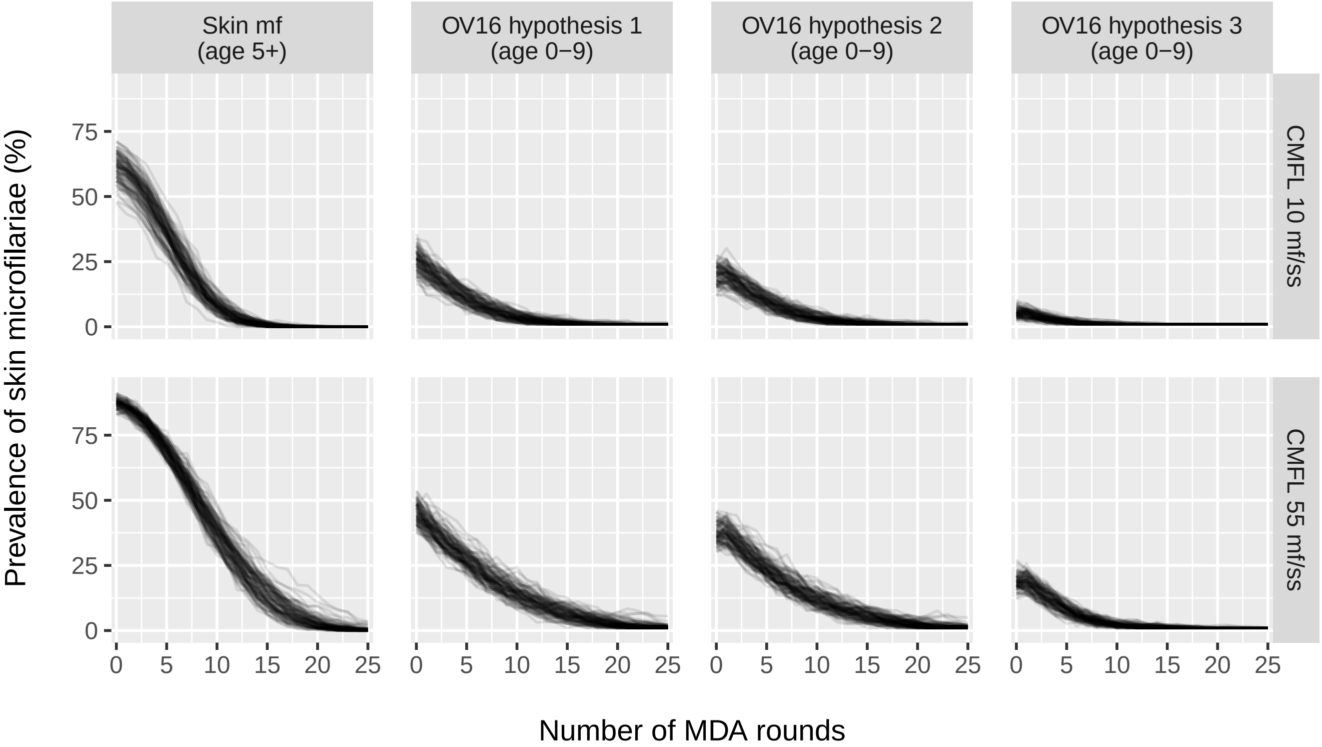

Skin mf
(age 5+)
OV16 hypothesis 1
(age 0−9)
OV16 hypothesis 2
(age 0−9)
OV16 hypothesis 3
(age 0−9)
CMFL 10 mf/ss
CMFL 55 mf/ss
Prevalence of skin microfilariae (%)
0
25
50
75
0
5
10
15
20
25
Number of MDA rounds
