# Supplementary material for: Modelling Anti-Ov16 IgG4 Antibody Prevalence as an Indicator for Evaluation and Decision Making in Onchocerciasis Elimination Programmes
Source: PLoS Negl Trop Dis. 2017 Jan 23;11(1):e0005314. doi: 10.1371/journal.pntd.0005314 (PMC5289624; doi:10.1371/journal.pntd.0005314)
Supplement: S3 Appendix — (PDF) [file pntd.0005314.s003.pdf]

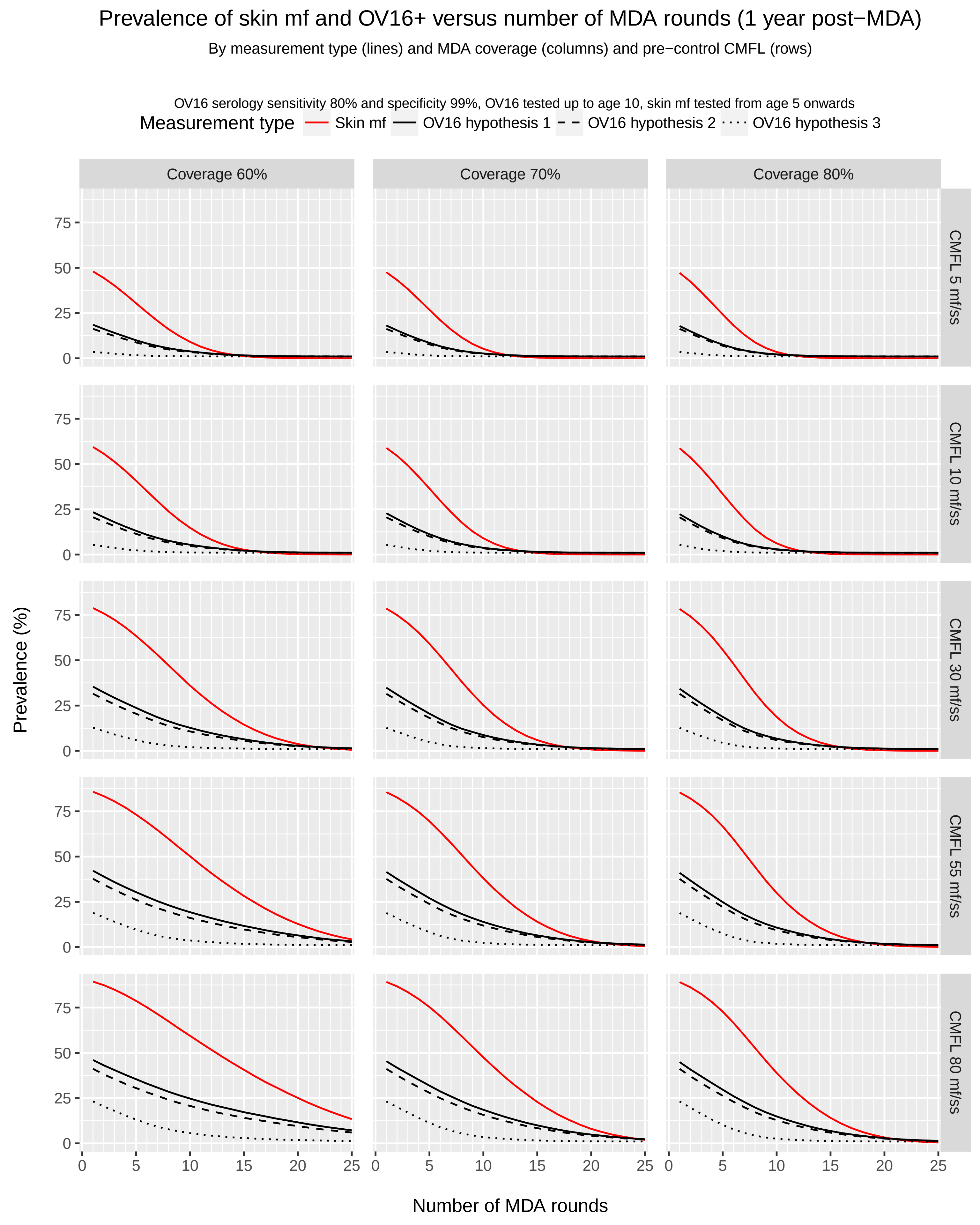
Prevalence of skin mf and OV16+ versus number of MDA rounds (1 year post−MDA)
By measurement type (lines) and MDA coverage (columns) and pre−control CMFL (rows)
OV16 serology sensitivity 80% and specificity 99%, OV16 tested up to age 10, skin mf tested from age 5 onwards
Measurement type
Skin mf
OV16 hypothesis 1
OV16 hypothesis 2
OV16 hypothesis 3
Coverage 60%
Coverage 70%
Coverage 80%
CMFL 5 mf/ss
CMFL 10 mf/ss
CMFL 30 mf/ss
CMFL 55 mf/ss
CMFL 80 mf/ss
Prevalence (%)
0
25
50
75
0
5
10
15
20
25
Number of MDA rounds

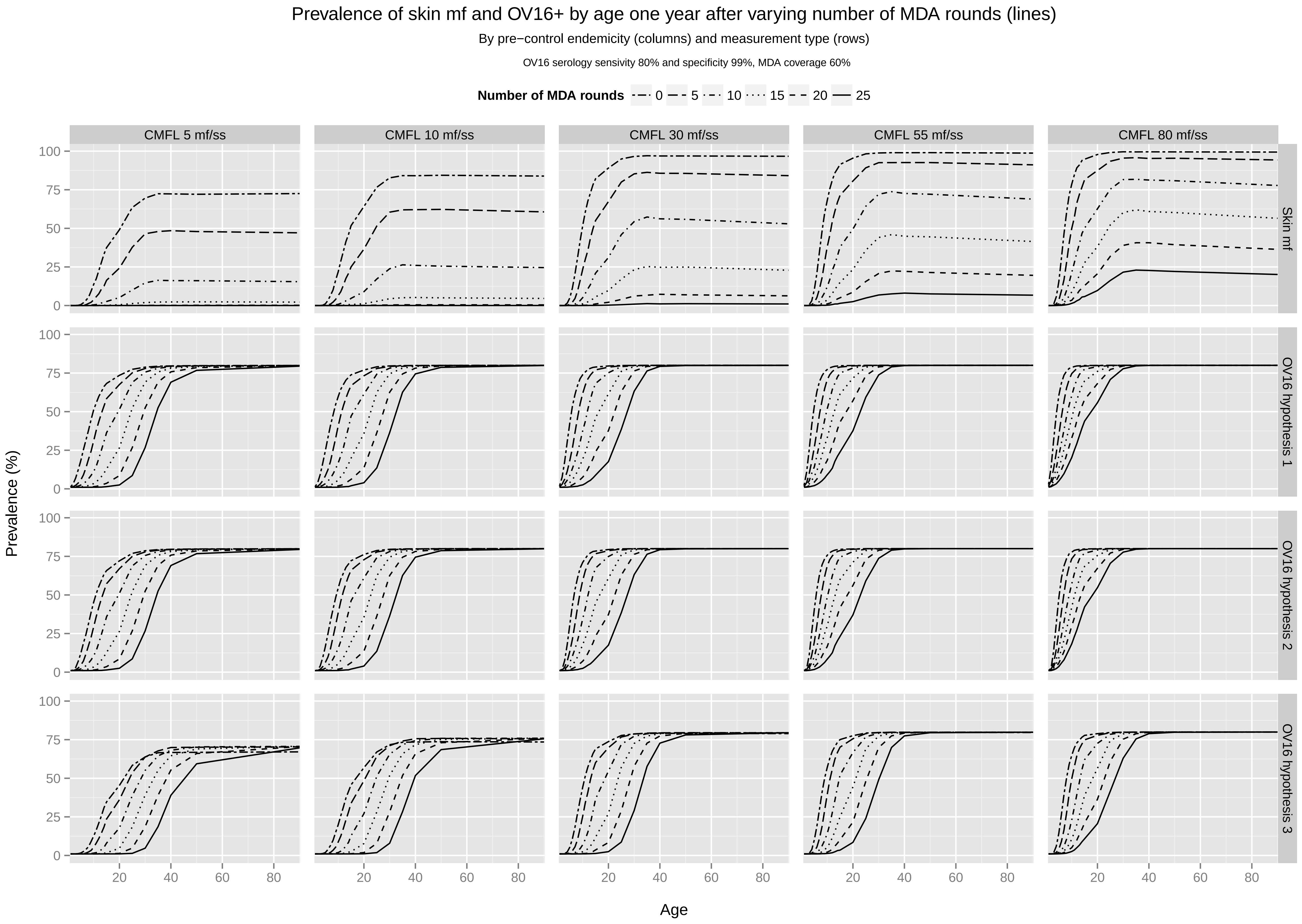
Prevalence of skin mf and OV16+ by age one year after varying number of MDA rounds (lines)
By pre−control endemicity (columns) and measurement type (rows)
OV16 serology sensivity 80% and specificity 99%, MDA coverage 60%
Number of MDA rounds
0
5
10
15
20
25
CMFL 5 mf/ss
CMFL 10 mf/ss
CMFL 30 mf/ss
CMFL 55 mf/ss
CMFL 80 mf/ss
Skin mf
OV16 hypothesis 1
OV16 hypothesis 2
OV16 hypothesis 3
Prevalence (%)
Age
0
25
50
75
100
20
40
60
80

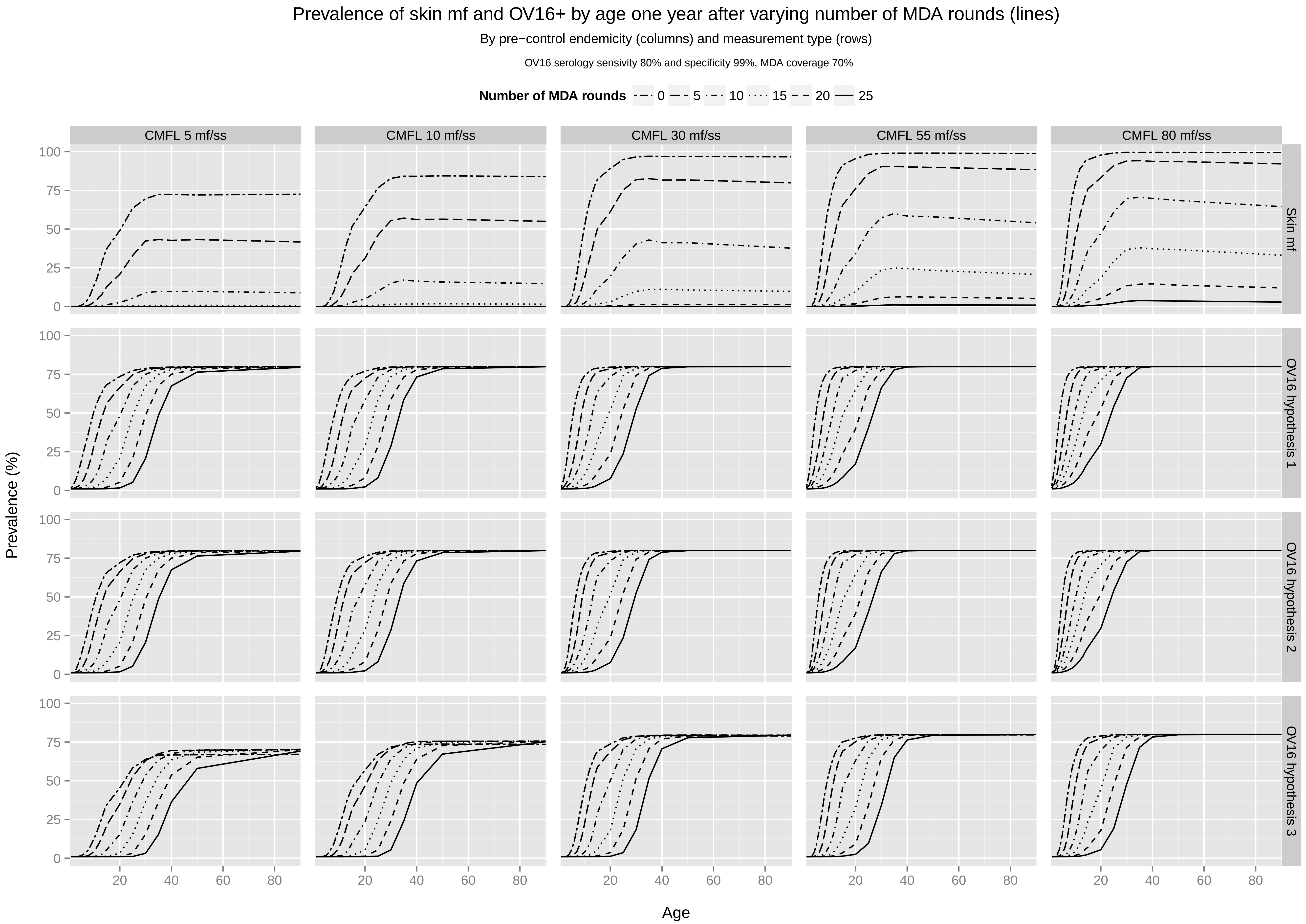

Prevalence of skin mf and OV16+ by age one year after varying number of MDA rounds (lines)
By pre−control endemicity (columns) and measurement type (rows)
OV16 serology sensivity 80% and specificity 99%, MDA coverage 70%
Number of MDA rounds
0
5
10
15
20
25
CMFL 5 mf/ss
CMFL 10 mf/ss
CMFL 30 mf/ss
CMFL 55 mf/ss
CMFL 80 mf/ss
Skin mf
OV16 hypothesis 1
OV16 hypothesis 2
OV16 hypothesis 3
Prevalence (%)
Age
0
25
50
75
100
20
40
60
80

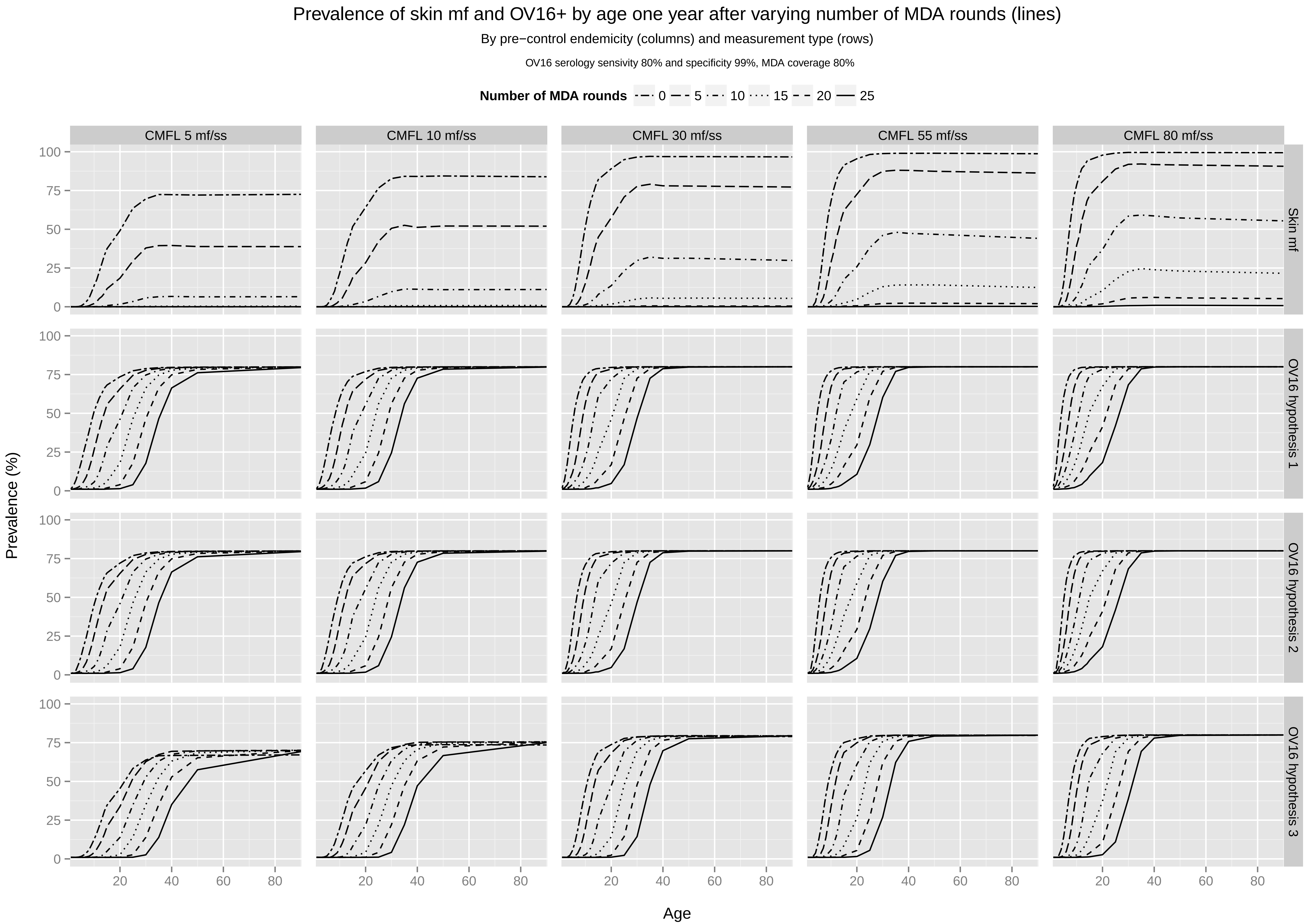

Prevalence of skin mf and OV16+ by age one year after varying number of MDA rounds (lines)
By pre−control endemicity (columns) and measurement type (rows)
OV16 serology sensivity 80% and specificity 99%, MDA coverage 80%
Number of MDA rounds
0
5
10
15
20
25
CMFL 5 mf/ss
CMFL 10 mf/ss
CMFL 30 mf/ss
CMFL 55 mf/ss
CMFL 80 mf/ss
Skin mf
OV16 hypothesis 1
OV16 hypothesis 2
OV16 hypothesis 3
Prevalence (%)
Age

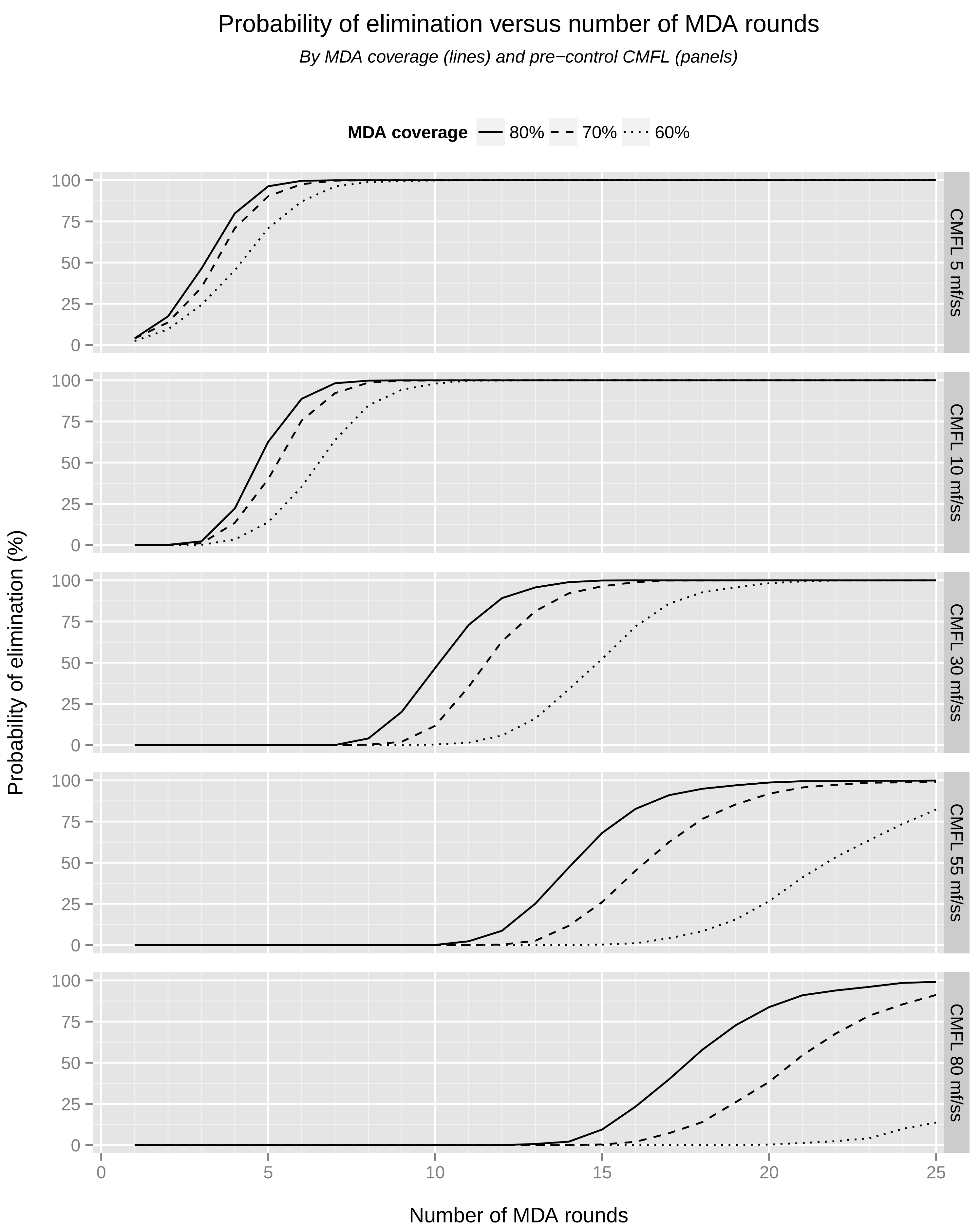
Probability of elimination versus number of MDA rounds
By MDA coverage (lines) and pre−control CMFL (panels)
MDA coverage
80%
70%
60%
CMFL 5 mf/ss
CMFL 10 mf/ss
CMFL 30 mf/ss
CMFL 55 mf/ss
CMFL 80 mf/ss
Probability of elimination (%)
Number of MDA rounds
0
25
50
75
100
0
5
10
15
20
25

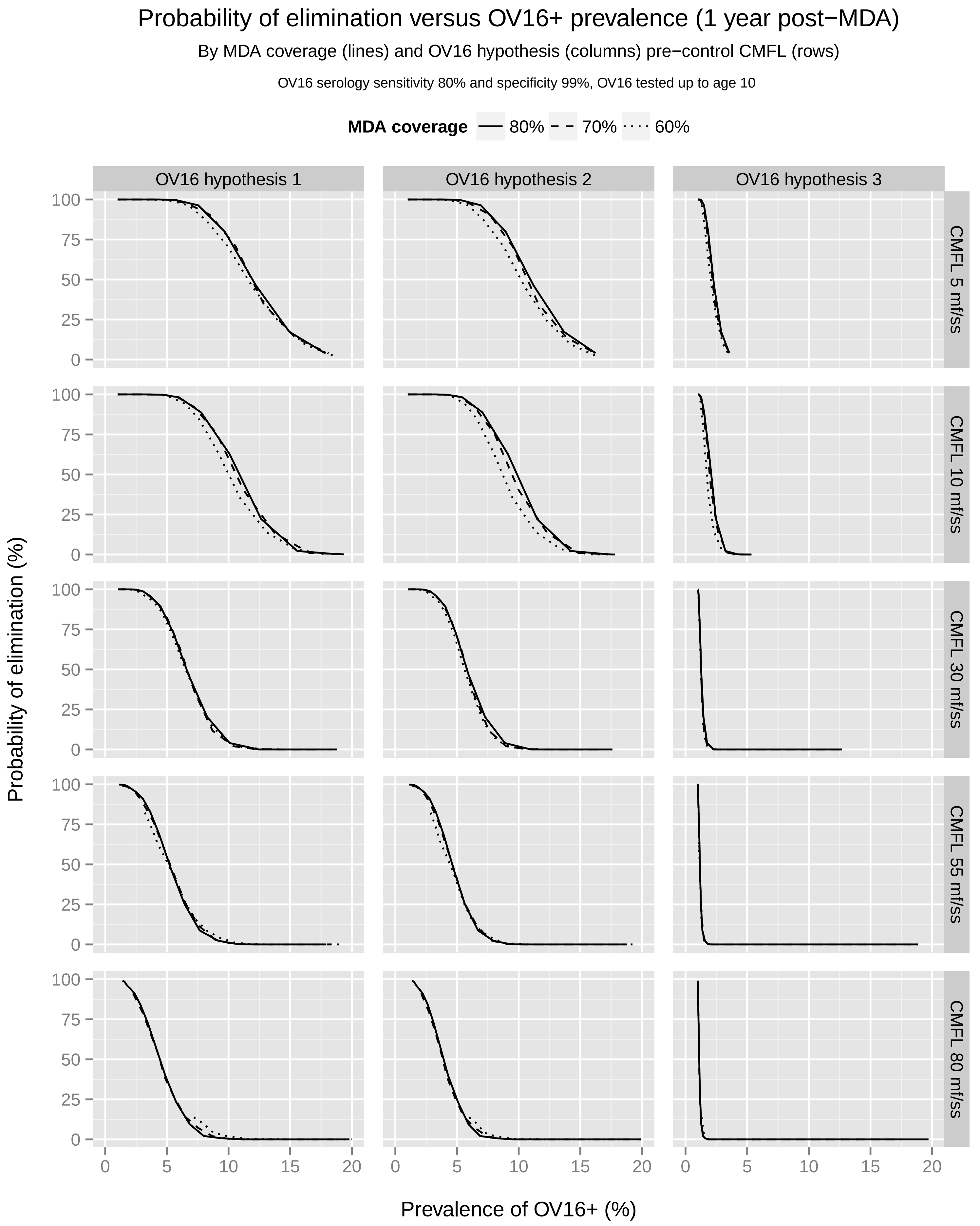
Probability of elimination versus OV16+ prevalence (1 year post−MDA)
By MDA coverage (lines) and OV16 hypothesis (columns) pre−control CMFL (rows)
OV16 serology sensitivity 80% and specificity 99%, OV16 tested up to age 10
MDA coverage
80%
70%
60%
OV16 hypothesis 1
OV16 hypothesis 2
OV16 hypothesis 3
CMFL 5 mf/ss
CMFL 10 mf/ss
CMFL 30 mf/ss
CMFL 55 mf/ss
CMFL 80 mf/ss
Probability of elimination (%)
Prevalence of OV16+ (%)
0
25
50
75
100
0
5
10
15
20

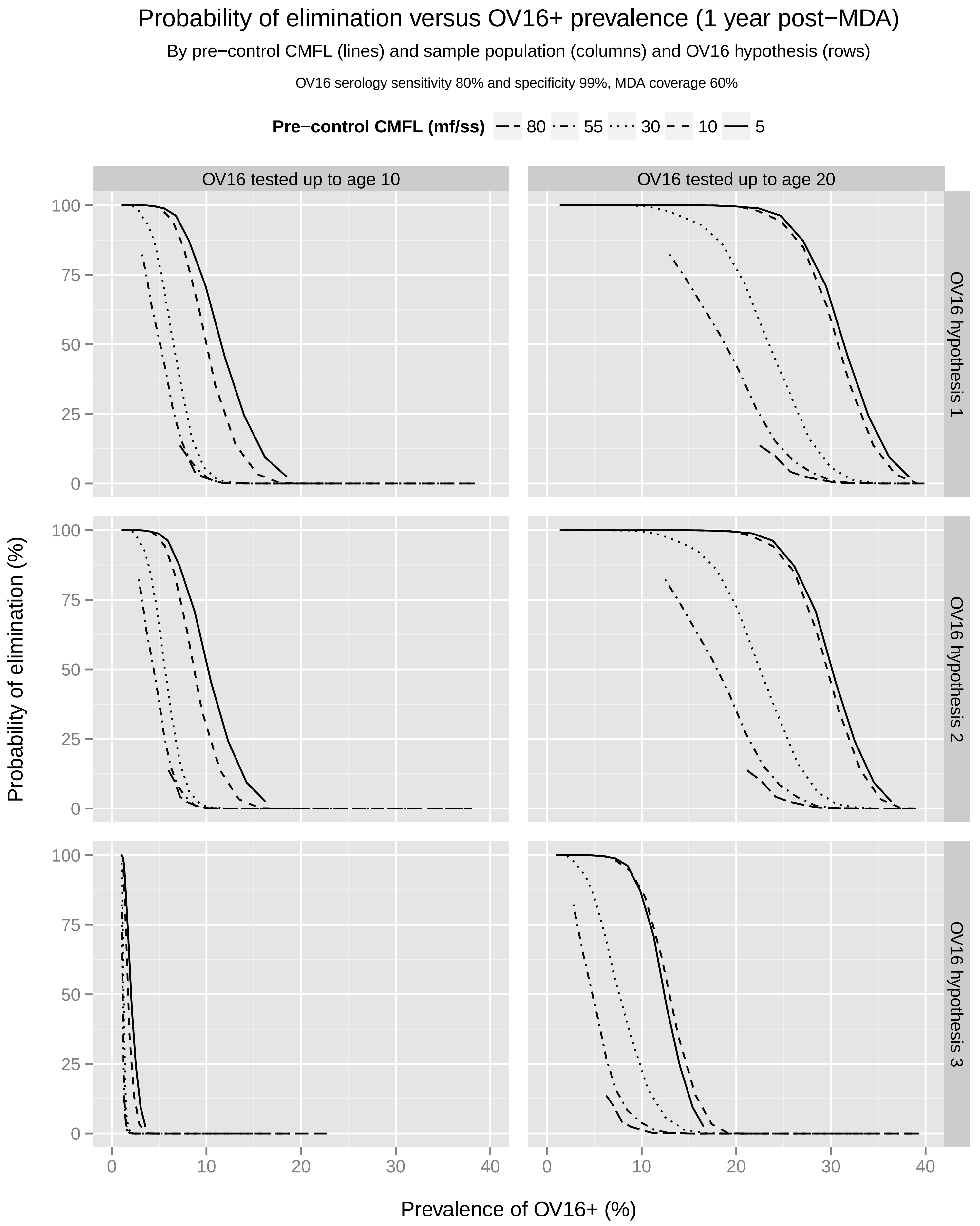
Probability of elimination versus OV16+ prevalence (1 year post−MDA)
By pre−control CMFL (lines) and sample population (columns) and OV16 hypothesis (rows)
OV16 serology sensitivity 80% and specificity 99%, MDA coverage 60%
Pre−control CMFL (mf/ss)
80
55
30
10
5
OV16 tested up to age 10
OV16 tested up to age 20
OV16 hypothesis 1
OV16 hypothesis 2
OV16 hypothesis 3
Probability of elimination (%)
Prevalence of OV16+ (%)
0
25
50
75
100
0
10
20
30
40

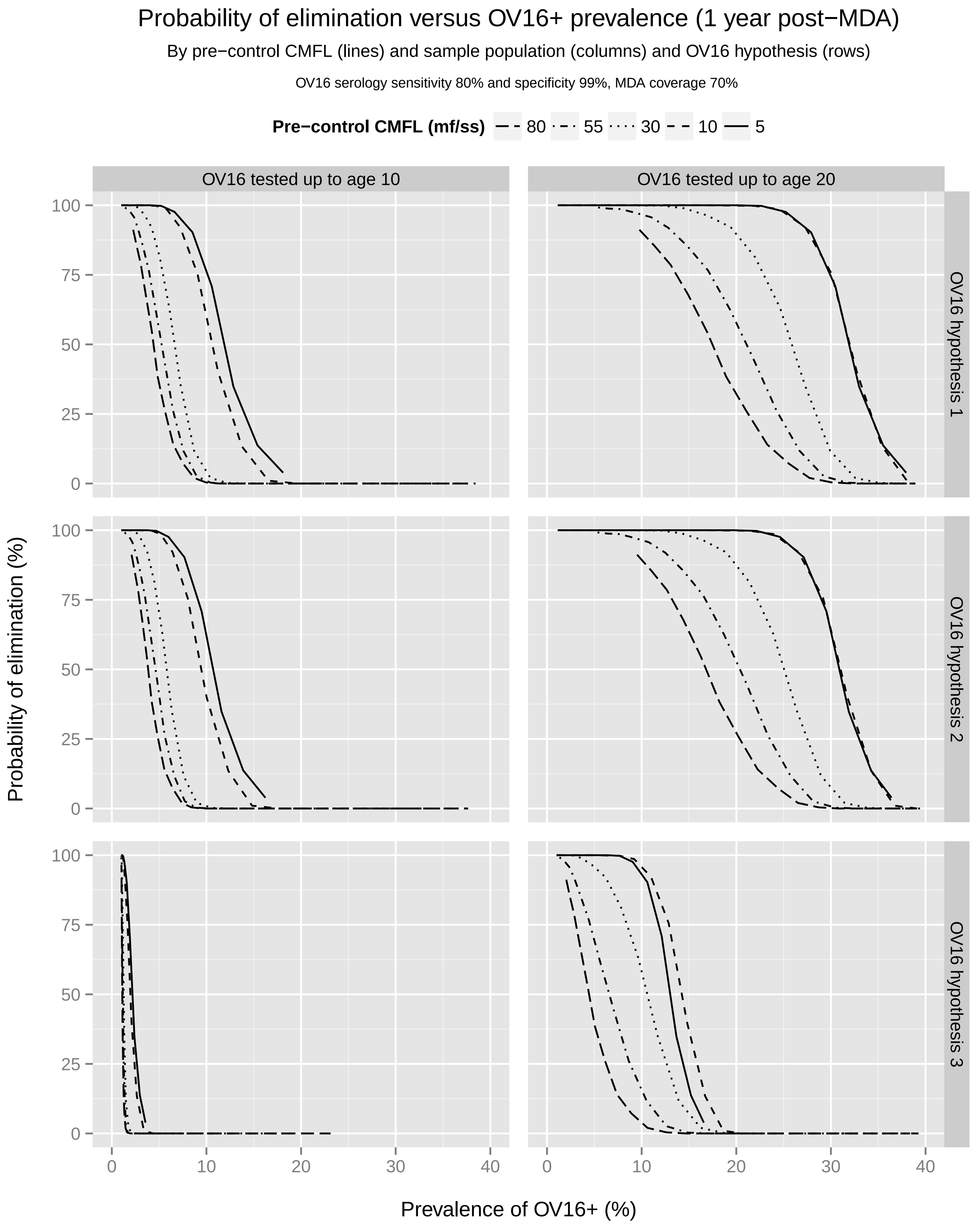

Probability of elimination versus OV16+ prevalence (1 year post−MDA)
By pre−control CMFL (lines) and sample population (columns) and OV16 hypothesis (rows)
OV16 serology sensitivity 80% and specificity 99%, MDA coverage 70%
Pre−control CMFL (mf/ss)
80
55
30
10
5
OV16 tested up to age 10
OV16 tested up to age 20
OV16 hypothesis 1
OV16 hypothesis 2
OV16 hypothesis 3
Probability of elimination (%)
Prevalence of OV16+ (%)
0
25
50
75
100
0
10
20
30
40

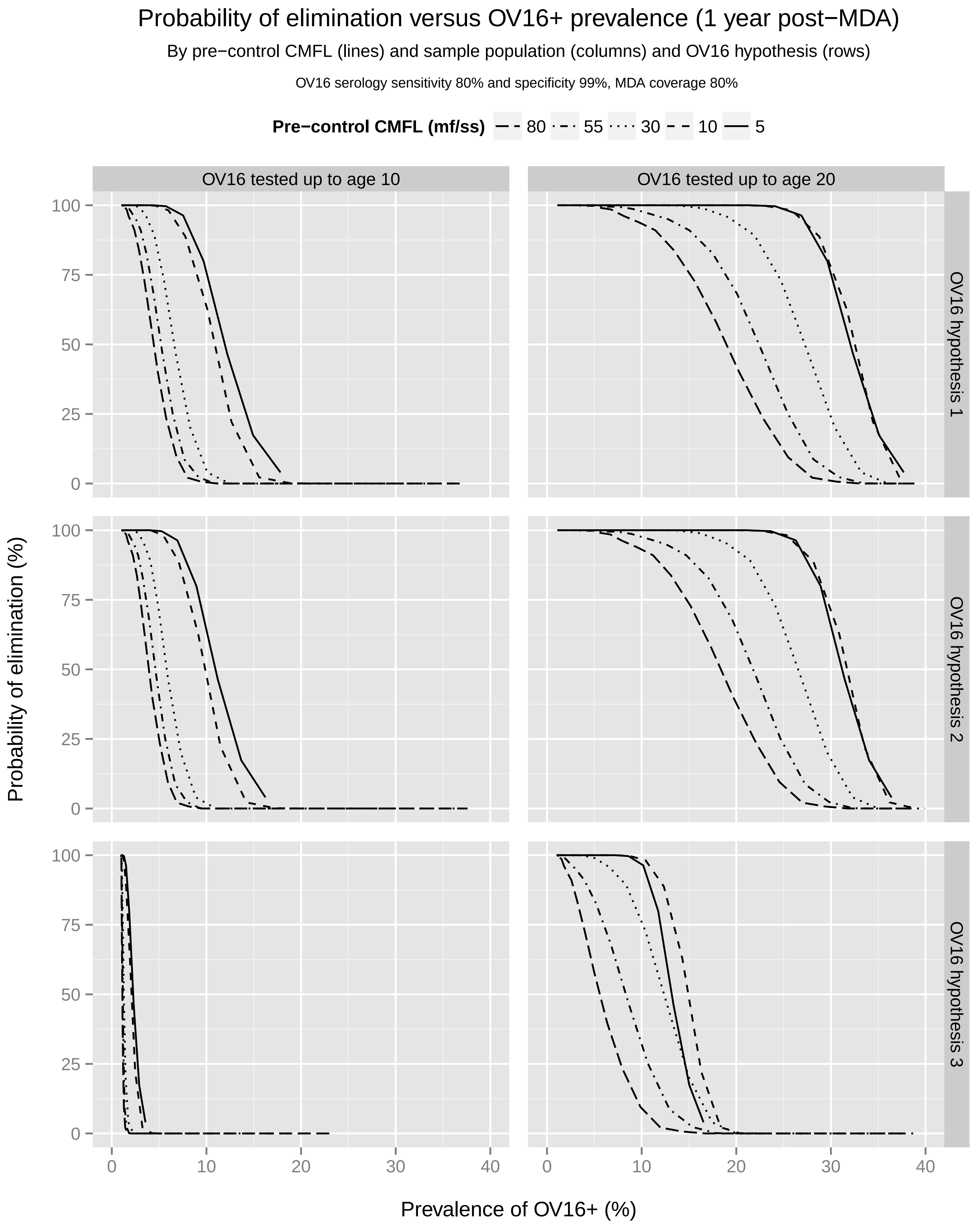

Probability of elimination versus OV16+ prevalence (1 year post−MDA)
By pre−control CMFL (lines) and sample population (columns) and OV16 hypothesis (rows)
OV16 serology sensitivity 80% and specificity 99%, MDA coverage 80%
Pre−control CMFL (mf/ss)
80
55
30
10
5
OV16 tested up to age 10
OV16 tested up to age 20
OV16 hypothesis 1
OV16 hypothesis 2
OV16 hypothesis 3
Probability of elimination (%)
Prevalence of OV16+ (%)
0
25
50
75
100
0
10
20
30
40
